# Brain-AI convergence: Generative world models and hierarchical attention for human intelligence

**DOI:** 10.1016/j.patter.2026.101593

**Published:** 2026-06-26

**Authors:** Shogo Ohmae, Keiko Ohmae

**Affiliations:** 1 Beijing Institute for Brain Research, Chinese Academy of Medical Sciences & Peking Union Medical College, Beijing 102206, China; 2 Beijing Key Laboratory of Brain Science and Brain-Machines Interface, Chinese Institute for Brain Research, Beijing (CIBR), Beijing 102206, China

**Keywords:** predictive coding, internal model, prediction-error learning, mirror neuron system, in-context learning, transformer, language processing, large language models, cerebellum, cortex

## Abstract

Recent advances in general-purpose AI provide new insights into how the neocortex and cerebellum, despite their uniform circuit architectures, support diverse functions and human intelligence. Beyond the traditional focus on visual processing, this paper offers a cross-domain comparison of the brain and AI through the lens of world-model-based computation. We argue that both the neocortex and cerebellum predict future world states from past inputs and construct predictive world models through prediction-error learning. These predictive world models are repurposed for sensory comprehension and motor output generation, thereby supporting multi-domain capabilities. Underlying these capabilities and even human-like adaptive intelligence, the neocortex implements hierarchical attention-based processing. Interestingly, autoregressive generative models in transformer-based AI have independently converged on similar computational principles. Together, these shared mechanisms suggest a common computational foundation through which uniform circuits can support cross-domain high-level intelligence in both biological and artificial systems.

## The bigger picture

Large language models (LLMs) now exhibit remarkable language abilities that are indistinguishable from those of humans in blind dialog tests, renewing the question of whether brain-AI similarity is limited to superficial, output-level resemblance or extends to internal computational processes. Previous comparative surveys have focused primarily on vision and have identified some parallels. In higher cognition and intelligence, however, the prevailing view has held that the underlying internal processes of the brain and AI remain fundamentally distinct. Against this background, we extend brain-AI comparison across sensory, motor, and cognitive domains, with particular emphasis on language processing as a representative domain of higher cognition. By comparing the neocortex, the cerebellum, and modern AI including LLMs, we identify four shared computational principles: (1) the formation of world models through prediction-error learning, (2) the reuse of those predictive models for both understanding and generation, (3) hierarchical attention-based processing, and (4) adaptive intelligence enabled by attention-based switching among expert modules. Together, the first two principles reframe the mirror-neuron concept as a biological mechanism for reusing predictive models for generation, paralleling autoregressive generation in LLMs.

Importantly, this paper moves beyond merely reaffirming partial similarities and instead argues for convergent evolution in the computational foundations underpinning higher cognition and intelligence in biological and artificial systems. The insights presented in this article bridge neuroscience and AI research, guide the design of more brain-inspired AI, and shed new light on the computational principles underlying the advanced intelligence characteristic of humans and AI.

## Graphical abstract

**Figure:**
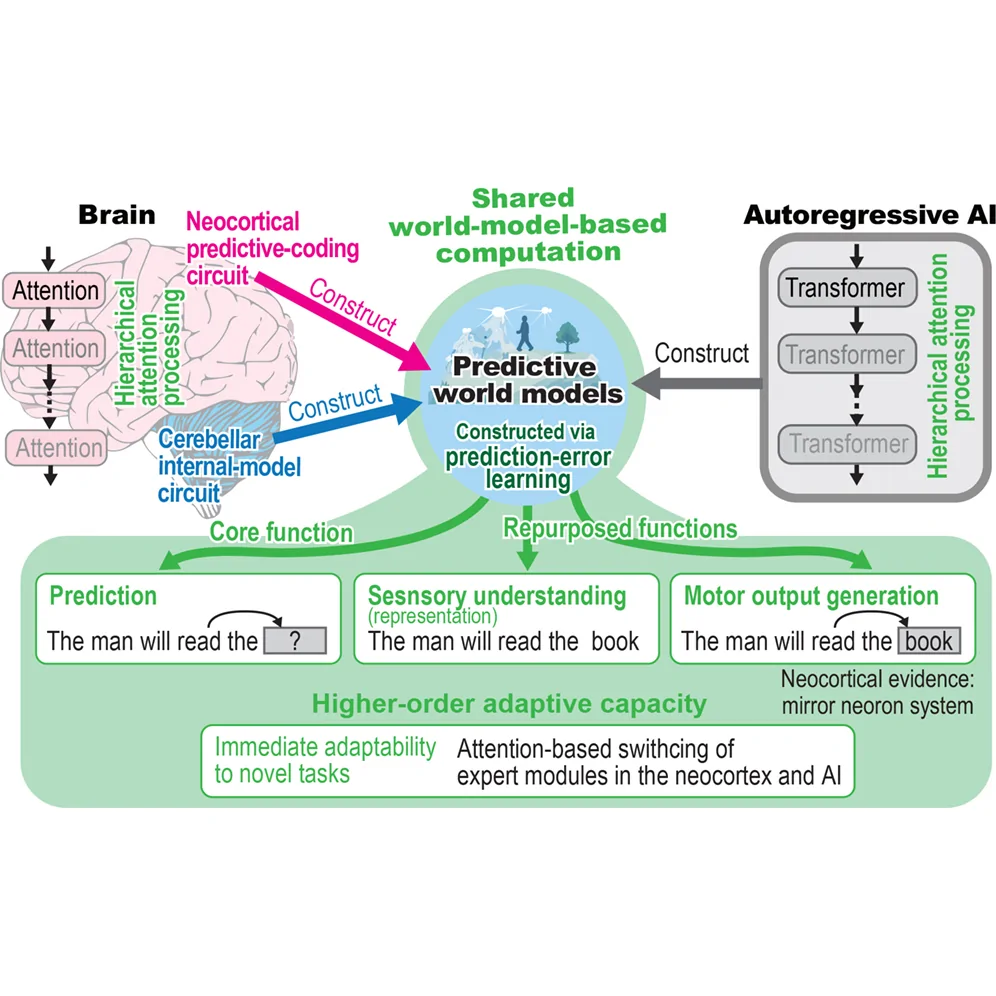

## Teaser

Large language models now exhibit human-like abilities, raising a fundamental question: are the brain and AI similar only at the surface level, or do they share deeper computational principles? This perspective argues that both systems build predictive world models through prediction-error learning and reuse those models for sensory understanding and motor generation. This indicates that the brain and AI share computational principles underlying advanced cognitive functions and intelligence, despite the long-standing view that their internal processes are fundamentally distinct.

## Introduction

Recent advances in AI have achieved performance comparable to that of the human brain across sensory, motor, and cognitive domains, highlighting the need for a comprehensive brain-AI comparison at the level of internal computation. In language processing, for example, the remarkably flexible and coherent responses generated by large language models (LLMs) such as ChatGPT were indistinguishable from human responses in blind dialogue tests,^1^ raising a fundamental question: does this brain-AI similarity reflect mere surface-level functional correspondence or does it point to deeper parallels in internal computational mechanisms? Examining such parallels is important for neuroscience, as it can advance ongoing efforts to use brain-like AI as a tool for probing neural processing and ultimately contribute to new theories and concepts.^2^^,^^3^^,^^4^^,^^5^^,^^6^^,^^7^^,^^8^^,^^9^^,^^10^^,^^11^^,^^12^^,^^13^ For AI research, systematically characterizing the similarities and differences between AI and brain processing can inform the design of more efficient and general-purpose brain-inspired architectures.

However, previous reviews comparing the brain and AI have focused primarily on visual processing, where brain-AI similarities are particularly prominent, leaving other functional domains only sparsely and unsystematically examined.^5^^,^^6^^,^^7^^,^^8^^,^^9^^,^^10^^,^^14^^,^^15^ In particular, even as AI systems have begun to exhibit higher-order cognitive capabilities—including language abilities long considered characteristic of humans—comprehensive comparative surveys of these functions have remained scarce. As a result, the conventional view has persisted that the cognitive functions underlying advanced intelligence are fundamentally distinct between the human brain and AI.^16^^,^^17^^,^^18^^,^^19^ To address this gap, this paper takes a broader approach, comparing the brain and AI systematically across sensory, motor, and cognitive domains. We emphasize that such a comparison should not be limited to simply enumerating similarities within each domain. The cerebral neocortex and cerebellum, which together comprise more than 99% of all cells in the human brain, achieve a wide range of functions through relatively uniform circuit structures and local circuit computations.^3^^,^^20^^,^^21^^,^^22^^,^^23^ Similarly, while early AI research produced many specialized single-function architectures, in recent years, general-purpose architectures capable of realizing multiple functions within a single circuit structure, most notably transformers, have become central to the field. Thus, in both the brain and AI, uniform circuits support functions across a wide range of domains, raising the possibility that brain-AI similarities may extend to the general-purpose mechanisms underlying diverse functions across domains.

To provide a comprehensive and structured comparison across domains, we break down circuit computation into three elements and analyze brain-AI similarities for each. (Throughout, we focus on computational mechanisms at the network level, defined by patterns of connectivity among neurons.) (1) Circuit structure: in the brain, the circuit structure is constrained by anatomical connectivity between neuron types and the synaptic sites responsible for plasticity and learning. In AI, by contrast, circuit architectures can be flexibly designed to meet task demands. In both cases, circuit structure critically determines the upper limit of a circuit’s computational capacity and information-transformation capability.^23^^,^^24^ (2) Input/output signals: the function of a circuit is characterized by the nature of its inputs and outputs. Even when the circuit structure is identical, differences in the properties of input/output signals can lead to substantial differences in function. (3) Circuit learning: even with similar input/output characteristics, different learning methods (e.g., supervised vs. unsupervised learning) can result in different intermediate representations across circuits.^25^^,^^26^^,^^27^^,^^28^^,^^29^^,^^30^^,^^31^

In the context of macro-scale computations beyond local circuits, such as whole-brain processing, world models have recently gained considerable attention as a shared concept between the brain and AI. A world model (including modality-specific models, such as language models and visual world models) is an internal representation of the external environment, or a virtual simulator of the world, constructed by the brain or AI. A world model is not merely a collection of individual instances, but rather comprises generalized representations, including abstract features and structural rules, that support generalization to novel situations.^9^^,^^28^^,^^32^ The concept of world models has developed independently in neuroscience and AI. In neuroscience, internal model theory, proposed in the 1970s, was established as a framework for explaining the predictive functions of the cerebellum in motor control, and has now become a standard framework for interpreting diverse cerebellar functions (see Kawato, Ohmae, et al.^33^).^23^^,^^34^^,^^35^^,^^36^^,^^37^^,^^38^^,^^33^^,^^39^^,^^40^^,^^41^^,^^42^^,^^43^^,^^44^^,^^45^^,^^46^ In the neocortex, the concept was introduced in the 2000s through research on predictive coding theory in sensory processing and model-based reinforcement learning.^4^^,^^21^^,^^47^^,^^48^^,^^49^^,^^50^^,^^51^^,^^52^^,^^53^^,^^54^^,^^55^^,^^56^^,^^57^^,^^58^^,^^59^ In AI, large-scale language models and world-model-based reinforcement learning have gained increasing attention as core technologies in the pursuit of artificial general intelligence (AGI). In this paper, we therefore position the world model concept as a central framework for systematic cross-domain comparison between the brain and AI.

This paper provides a comprehensive comparison of circuit computations in the brain and AI through the lens of world-model-based processing. We place particular emphasis on language processing as a cognitive function that encompasses both sensory and motor processing. Based on the comparison between the mirror-neuron concept in neuroscience and autoregressive generation in AI systems (such as GPT), we identify a broad convergence: the neocortex, cerebellum, and AI all construct predictive world models through prediction-error learning, use the abstracted and compressed representations formed within these models to understand the sensory world, and repurpose their predictive outputs for motor output generation. This reuse enables multifunctional processing from sensory to motor domains. We further argue that the neocortex and transformer-based language AI exhibit key parallels, including hierarchical attention-based processing and adaptive intelligence enabled by attention-based switching among expert modules. The approach and insights presented in this paper provide a promising foundation for future research bridging neuroscience and AI, with the aim of understanding human-like general intelligence.

## Brain-AI comparison in sensory processing

### Sensory information processing in the neocortex

**Figure 1:**
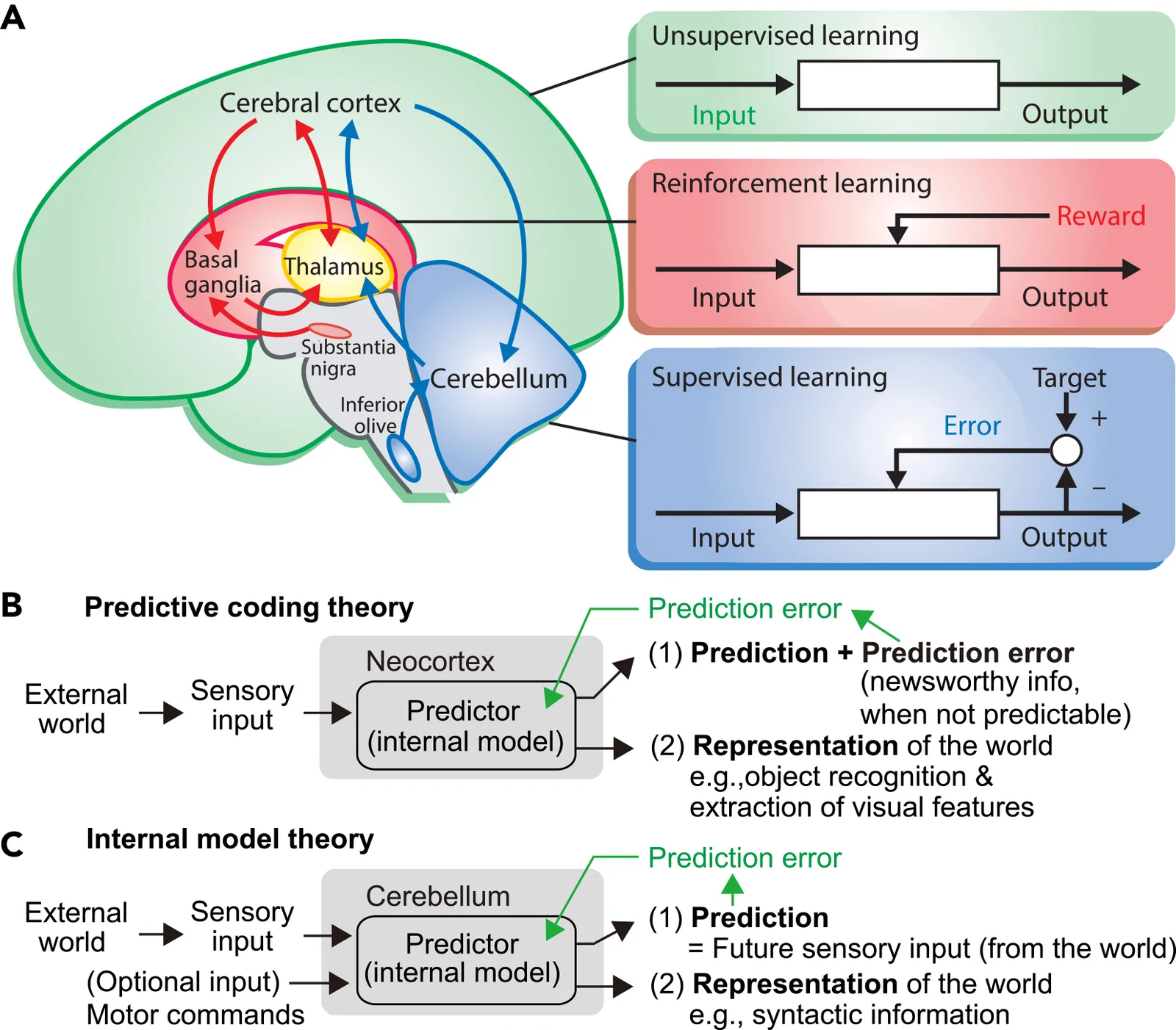
Theoretical frameworks of learning and processing in the neocortex and cerebellum (A) Unsupervised learning theory of the neocortex. Neocortical processing is established through unsupervised learning (green). The input and output signals of the neocortex are essentially the same, with learning driven by minimizing reconstruction error. In contrast, the cerebellum relies on supervised learning (blue), receiving high-level supervisory signals (“target”) from other brain regions. In supervised learning, the training target is already processed signals, and the circuit is trained to match its output to that target. In unsupervised learning, by contrast, the training target is often not itself meaningful as in input reconstruction. Instead, meaningful representations emerge in intermediate layers through information compression and feature extraction, and these representations constitute the effective output of information processing. Adapted from Doya (2000), with permission from Elsevier. (B) Predictive coding theory. The neocortex functions as a predictor, continuously receiving sensory inputs and (1) generating predictions of future inputs while outputting prediction errors as “newsworthy” signals. When the input matches the prediction, the output is zero, as the information carries no news value; only unpredicted inputs give rise to newsworthy signals. The prediction errors, in turn, serve as learning signals to update the predictor (green). Additionally, through information compression and feature extraction, this predictor (2) generates abstract representations of the external world, thereby supporting perceptual understanding (e.g., visual feature extraction, object recognition, and 3D reconstruction). (C) Internal model theory of the cerebellum. The cerebellum also functions as a predictor, receiving inputs from the external world, (1) generating predictions of future inputs, and learning from prediction errors. Accumulating evidence indicates that the learning target in the cerebellum is the future sensory input itself, rather than a high-level supervisory signal. Point (2) is detailed in Figure 3B.

Research on biological sensory processing, particularly in the visual system, has led to influential theoretical proposals regarding input/output signals and learning algorithms in the neocortex. A prominent example is the unsupervised learning theory of the neocortex, proposed in 1999 (Figure 1A, green).^60^ This theory builds on the finding that processing in the primary visual neocortex can be explained by information compression performed by a sparse autoencoder trained via unsupervised learning and has been substantiated by experimental evidence (Figures S1A and S1B).^7^^,^^14^^,^^29^^,^^60^^,^^61^^,^^62^^,^^63^ In addition, the hierarchical processing in the neocortex (e.g., in vision, the primary visual neocortex detects local edges and higher-order neocortices extract abstract features such as human faces) can be explained by a deep-layered sparse autoencoder (Figure S1C).^64^^,^^65^^,^^66^^,^^67^ More recently, convolutional neural networks (CNNs), which are computationally more efficient than sparse autoencoders, have been used to approximate local circuits in the neocortex.^5^^,^^6^^,^^7^^,^^8^^,^^9^^,^^10^^,^^14^^,^^15^^,^^62^^,^^63^^,^^68^^,^^69^^,^^70^^,^^71^^,^^72^^,^^73^^,^^74^

Despite these successes, autoencoders and CNNs are feedforward circuit architectures and therefore fail to capture the recurrent structure of the neocortical circuit, in which diverse cell types exhibit complex recurrent connectivity that enables the integration of past and present information.^75^^,^^76^^,^^77^^,^^78^ Consequently, although these feedforward-based theoretical accounts of the neocortex can explain static sensory processing (e.g., still image processing), they are insufficient to account for dynamic sensory processing (e.g., video processing).^23^^,^^76^^,^^79^^,^^80^^,^^81^^,^^82^^,^^83^^,^^84^^,^^85^^,^^86^^,^^87^^,^^88^^,^^89^ To address this limitation, the framework of neocortical unsupervised learning has evolved into the theory of predictive coding (Figure S2),^52^^,^^90^^,^^91^^,^^92^ which is now supported by abundant experimental evidence.^12^^,^^91^^,^^93^^,^^94^^,^^95^^,^^96^^,^^97^^,^^98^^,^^99^ According to predictive coding theory, the neocortex functions as a “predictor” of incoming sensory inputs from the external world, transforming these inputs into predictions and prediction errors (Figure 1B).^52^ Among these, the prediction error serves as a “newsworthy signal” and plays a central role in information processing. This prediction error is also used for learning in the predictor (i.e., the learning remains unsupervised). At the local circuit level, the neocortex has been approximated by recurrent neural networks (RNNs) such as long short-term memory (LSTM) networks, and their biological plausibility has been actively explored.^100^^,^^101^ Within predictive coding circuits, intermediate layers perform information compression and feature extraction.^75^^,^^77^^,^^91^ Moreover, the hierarchical structure of deep-layered predictive coding circuits enables the progressive extraction and prediction of information at longer time scales and higher levels of abstraction (Figures S2B–S2D).^4^^,^^21^^,^^91^^,^^92^^,^^102^

Predictive coding is one of the most well-established computational theories of the neocortex. At the macro level, this framework posits that neocortical processing has two major functions (Figure 1B): (1) generating predictions and prediction errors in response to sensory inputs, and acquiring a predictor of the sensory world—that is, an internal world model—through unsupervised prediction-error learning and (2) forming abstract representations of the external world as components of this internal model, thereby enabling sensory comprehension.^4^^,^^21^^,^^48^^,^^49^^,^^54^^,^^55^^,^^56^^,^^57^^,^^58^

### Cerebellar sensory processing through internal world models

Early cerebellar research focused primarily on motor control. In this context, internal model theory was proposed, positing that the cerebellum implements an internal model that predicts future world states and the resulting sensory inputs on the basis of current sensory inputs and motor commands (Figure 1C), thereby providing virtual feedback about movement outcomes to compensate for delays in actual sensory feedback. This theory was later extended to non-motor contexts, in which the cerebellum is thought to predict future sensory inputs on the basis of current sensory inputs. Extensive experimental evidence supports this framework.^23^^,^^34^^,^^35^^,^^36^^,^^37^^,^^38^^,^^33^^,^^39^^,^^40^^,^^41^^,^^42^^,^^43^^,^^44^^,^^45^^,^^46^ According to this theory, cerebellar learning is driven by prediction-error learning. From this perspective, the cerebellar input/output signals and learning can be regarded as functionally equivalent to those of the neocortex.

Traditionally, the cerebellum has been considered a supervised learning system (Figure 1A, blue), whereas the neocortex has been viewed as an unsupervised learning system.^7^^,^^14^^,^^29^^,^^43^^,^^44^^,^^60^^,^^61^^,^^62^ However, the cerebellar teaching signals are simply prediction errors and do not necessarily contain highly processed information, such as the ground-truth labels used in supervised machine learning.^43^^,^^44^^,^^45^^,^^46^^,^^103^^,^^104^^,^^105^^,^^106^ Thus, cerebellar learning can be reformulated as unsupervised prediction-error learning, analogous to learning in the neocortex and in self-supervised machine learning.

This reformulation, however, does not negate the implementation-level foundations of cerebellar learning. In contrast to the neocortex, the mechanistic basis of prediction-error learning is well established in the cerebellum (Figure S3). First, prediction-error signals are generated in the inferior olive by comparing the cerebellar predictive outputs with actual sensory signals and are fed back to Purkinje cells and cerebellar nuclear neurons via the olivo-cerebellar pathway. Second, as a credit-assignment mechanism for learning, synaptic weights associated with sensory parallel-fiber inputs arriving at Purkinje cells immediately before the prediction-error signal are selectively weakened.^103^^,^^107^ These implementation-level mechanisms remain valid even when the cerebellum is reconceptualized as an unsupervised learning machine.

### Complementary roles of the neocortex and cerebellum

Although both the neocortex and cerebellum acquire world models through prediction-error learning, they differ in processing speed, temporal integration, and learning speed. Because cerebellar circuitry is simpler than neocortical circuitry (Figure S3), the cerebellum is relatively well suited for rapid computation, even on the millisecond timescale,^108^^,^^109^^,^^110^ a property that is likely particularly essential in motor control. This speed advantage may allow the cerebellum to provide predictive support for ongoing neocortical processing.^22^^,^^103^^,^^111^ In temporal integration, the neocortex implements attention-based processing, which flexibly integrates past information in a more context-dependent manner, thereby potentially generating more accurate predictions (see the section “hierarchical attention-based processing as the core of neocortical processing”), whereas the cerebellum relies on non-attentional processing, with a fixed weighting of immediately past inputs (Figure 3). In learning, the cerebellum acquires predictions more rapidly,^112^^,^^113^^,^^114^ which can in turn facilitate neocortical learning and retention.^115^^,^^116^ From a world-model perspective, these differences suggest that the cerebellum rapidly acquires coarse world models and generates fast outputs, thereby supporting the slower learning and slower computation of more refined world models in the neocortex. This interpretation is consistent with clinical observations that cognitive deficits following cerebellar damage are broader and more persistent during development than in adulthood,^117^^,^^118^^,^^119^^,^^120^^,^^121^^,^^122^^,^^123^^,^^124^ owing to disrupted growth of the connected neocortex.^118^^,^^119^^,^^125^

## Brain-AI comparison in language processing

Language processing encompasses both “language comprehension,” in which words are recognized, word sequences are processed according to grammatical rules, and ultimately representations of semantic content are generated, thereby enabling understanding of sentence meaning,^99^^,^^126^^,^^127^^,^^128^^,^^129^^,^^130^^,^^131^^,^^132^^,^^133^ and “language generation,” in which word sequences are planned and produced. In this sense, it spans both sensory and motor domains, making it particularly well suited for cross-domain comparisons.

## Sensory language processing

### Neocortical language processing through hierarchical predictive coding

Like visual processing, the neocortical language pathway is also hierarchically organized, with information flowing from lower-order language areas (e.g., superior temporal sulcus) to higher-order language areas (e.g., inferior frontal gyrus, angular gyrus, and supramarginal gyrus). In contrast to the sensory neocortex, for which unsupervised learning has been proposed, language acquisition in the brain has historically been understood through innate language theory, which holds that core language functions are innate and genetically determined.^134^^,^^135^^,^^136^ However, recent computational neuroscience has challenged this view, demonstrating that language functions that were previously assumed impossible to learn can in fact be acquired through postnatal biological experience and unsupervised learning.^137^^,^^138^ Reflecting this shift, empirical research on human neocortical language processing has advanced dramatically since 2021, including fMRI and single-neuron electrophysiological studies, and has yielded two important insights. First, neocortical language processing can be explained by extending the hierarchical predictive coding framework of sensory processing to language input. Specifically, neocortical signals during language comprehension contain both prediction signals and prediction error signals for the next word,^139^^,^^140^ and these predictions are organized hierarchically, with higher-order language areas making more abstract predictions, such as predicting the semantic category of the next word.^99^^,^^141^^,^^142^ This extraction of increasingly abstract language information aligns naturally with the theoretical framework of hierarchical predictive coding in language.^4^^,^^59^^,^^143^ Second, predictive coding theory predicts that neocortical signals should resemble those of hierarchical language AI trained on next-word prediction; indeed, neocortical signals most closely resemble such AI systems when compared against other language AI systems.^130^^,^^139^ Furthermore, neocortical signals are better explained by a modified next-word-predicting AI incorporating the predictive coding framework.^99^

Collectively, these empirical findings indicate that neocortical language processing operates as hierarchical predictive coding, with lower layers performing simple word prediction and higher layers predicting more abstract linguistic content. From the perspective of the world model, through prediction-error learning, the neocortex acquires a language model that encodes abstract linguistic information and uses this model for prediction, representation, and comprehension (as in Figure 1B).

### Large-scale language models in AI

**Figure 2:**
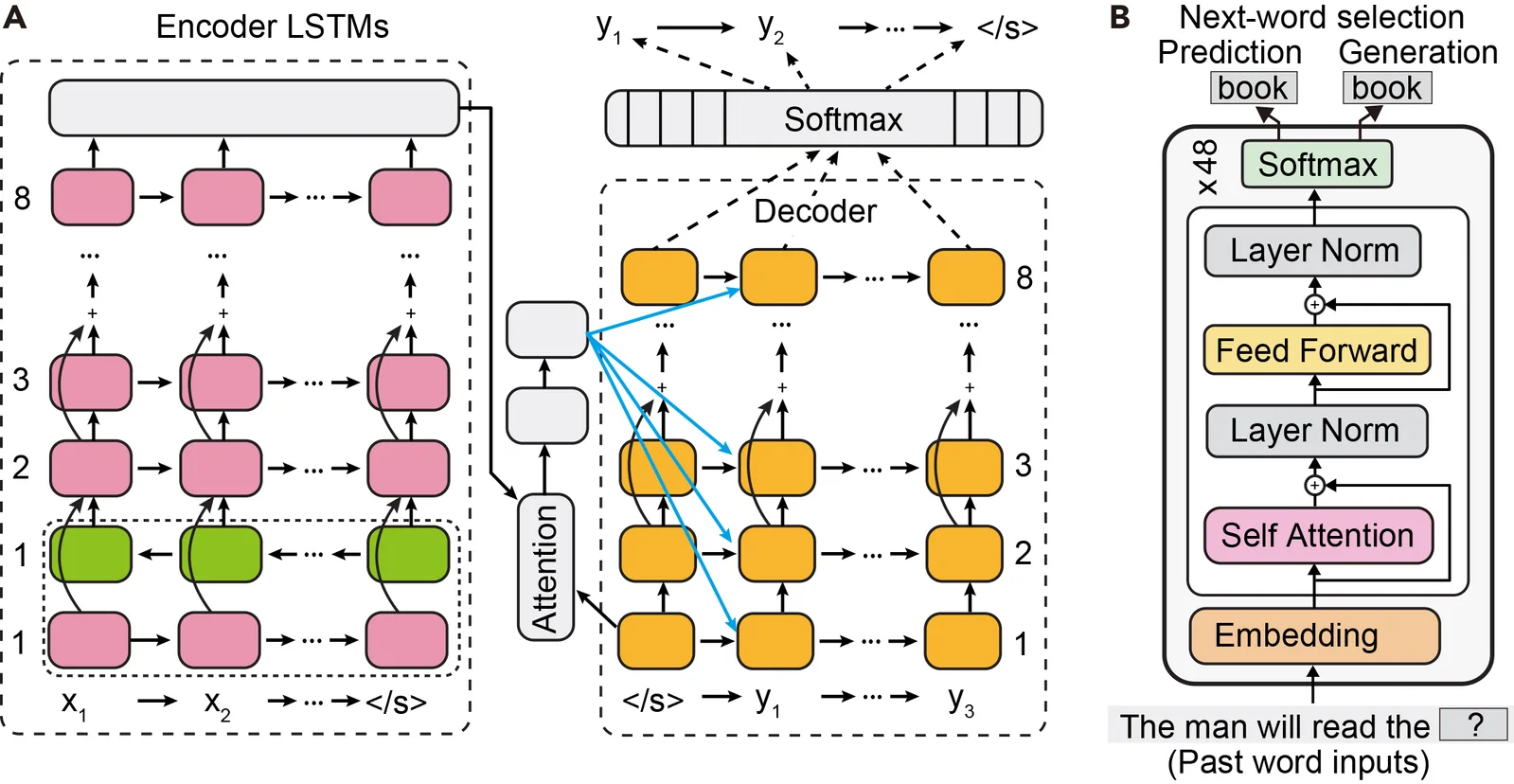
Language-processing AI and analogy to the neocortex (A) The translation circuit of Google Neural Machine Translation (GNMT). This circuit consists of encoder, attention, and decoder units, with the encoder-decoder combination used only once throughout the entire circuit. Left: the encoder is a stacked-LSTM circuit that sequentially receives the words of a source sentence and integrates them to generate the signal that represents the meaning of the entire sentence. The encoder has eight layers (one bidirectional layer plus seven unidirectional layers), and the outputs of the final layer are combined to generate a vector representing the meaning of the entire sentence. The bidirectional layer ensures that the representation for even the first words can include the context of the sentence. Center: the attention unit is a two-layer neural network that receives the most recently translated word from the decoder (arrow from the decoder; only the signal flow during the second word [y2] translation is illustrated) and the vector representation of the source sentence from the encoder (arrow from the encoder), and then creates a weight-coefficient signal, indicating which word in the source sentence should be focused on (i.e., the additive attention). The attention unit then provides this weight-coefficient multiplied source-sentence signal to each layer of the decoder (blue arrows to the decoder). The decoder is an eight-layer LSTM that sequentially generates the probability of each word being the next translated word. The circuit is trained through supervised learning with manually constructed translation corpora as the correct targets. The error signal, calculated by comparing the generated word with the correct word, is delivered to the entire encoder-decoder system via backpropagation to update the synaptic weights. Adapted from Ramsundar & Zadeh (2018), with permission from O'Reilly Media. (B) Transformer circuit for GPT-style language AI. Since GPT is a circuit that predicts the next word, it does not have an encoder-decoder structure like GNMT but has an integrated structure. During training, GPT receives a sequence of preceding words and predicts the next word (e.g., book). After training, for sentence generation, the input is an unfinished sentence created immediately before by GPT, and GPT repurposes the same output system to generate the next word. Circuit: repeating blocks mainly consist of an attention unit and a two-layer feedforward neural network, with these blocks stacked 12 times in GPT-1 and 48 times in GPT-2. Input/output: the input is a word sequence in a sparse representation (the maximum text length is 2,048 words). The output is a probability representation of the next word candidate. Learning: unsupervised learning through next-word prediction. Adapted from Wu et al. (2024)^148^ (CC BY 4.0), with elements from Radford et al. (2018).^149^

To examine the similarities between language AI and the neocortex, we next trace the development of language AI systems. A major early milestone in this trajectory was Google Neural Machine Translation (GNMT), which was adopted by Google Translate in 2016 (Figure 2A).^144^^,^^145^ The GNMT circuit primarily consists of a deep-layered RNN incorporating LSTM. GNMT relies on supervised learning with human-translated sentences, and generates errors only in the final layer, propagating them to all layers by backpropagation. While GNMT differs from the neocortex in these respects (neocortical learning is unsupervised, and prediction errors in predictive coding are generated and consumed at each layer), it is noteworthy that the GNMT encoder can convert a sentence in one language into a semantic vector (the population activity of a group of neurons), and this vector contains sufficient semantic information for the decoder to generate a sentence with the same meaning in another language. This demonstrates that deep-layered RNNs can convert sentences into semantic representations encoded as population-coded vectors, an intriguing finding from a neuroscience perspective.^146^^,^^147^

After GNMT, due to limitations in computational cost and speed (low efficiency of parallel processing with GPUs), AI language processing shifted from sequentially inputting words into an RNN to feeding entire sentences into a feedforward circuit known as a transformer.^150^^,^^151^ Although this input format differs from that of the brain, the transformer expands the input dimensions to include the time axis, enabling the integration of information over time, similar to an RNN. Notably, the prevailing learning method also transitioned from supervised to unsupervised learning, thereby bringing AI learning closer to learning in the brain.

The first system to demonstrate the power and versatility of unsupervised learning in a transformer circuit was Google’s BERT (bidirectional encoder representations from transformers).^26^ Pre-BERT language AIs were limited by the lack of versatility in supervised learning (i.e., accuracy dropped significantly outside of their trained tasks). To overcome this limitation, BERT, with an encoder-decoder circuit, employed large-scale unsupervised learning of masked-word completion. Subsequently, by replacing the decoder part with a small task-specific output layer, supervised learning of only that layer was sufficient to achieve state-of-the-art performance. The success of this method on a wide range of language tasks indicates that the encoder part of BERT is capable of generalized language comprehension sufficient for diverse tasks.

Later, the power of unsupervised prediction-error learning was strikingly demonstrated by the emergence of the GPT (generative pre-trained transformer) series, including ChatGPT (Figure 2B).^148^^,^^149^^,^^152^ Since GPT-2 in particular, GPTs have been extensively trained on next-word prediction. Remarkably, GPTs can repurpose their predictive outputs for next-word selection during text generation. This capability enables GPTs to generate coherent sentences and produce textual responses across a wide range of language tasks with state-of-the-art performance, without the task-specific supervised fine-tuning required for BERT.

All of these language AI systems can appropriately interpret previously unseen sentences with complex syntax. This suggests that they acquire language models that capture grammatical rules and syntactic structure and use those models to form representations of sentence meaning, thereby enabling language comprehension. Among them, GPT is particularly notable because its language model is acquired through prediction-error learning, analogous to learning in the brain.^130^^,^^138^^,^^139^^,^^140^^,^^142^ Indeed, GPT’s signals more closely resemble neocortical signals than do those of BERT or GNMT.^139^

### Computational parallels between the neocortex and transformer circuits

In light of these strong parallels between GPT and the neocortex, we next examine neocortical and transformer processing, focusing on both their parallels and differences (see Figure S4 for the circuit design and processing of the transformer).

At least four major parallels can be identified. First, both the neocortex and transformers excel at integrating information over time. Second, both are circuits with relatively uniform structures yet remarkable versatility. Transformer circuits have achieved state-of-the-art performance in language processing, vision, and video generation (e.g., vision transformer and DALL-E), suggesting their potential as a general-purpose architecture,^28^^,^^150^^,^^153^^,^^154^ much like the neocortex.^20^^,^^23^ Third, through large-scale prediction-error learning, powerful world models can be built by both the neocortex^4^^,^^21^^,^^47^^,^^48^^,^^49^^,^^50^^,^^51^^,^^52^^,^^53^^,^^54^^,^^55^^,^^56^^,^^57^^,^^58^^,^^59^ and transformers.^149^^,^^151^^,^^152^^,^^154^^,^^155^ Fourth, both possess attentional processing mechanisms. Transformer circuits were designed with reference to the attention mechanisms of the neocortex,^24^^,^^156^ particularly top-down attention to spotlight specific information depending on context. Taken together, the parallels between the neocortex and transformer circuits lie primarily at the algorithmic level: both acquire models of the external world through prediction-error learning and achieve context-dependent integration of information over time through attention mechanisms.

By contrast, the major differences lie at the implementation level, particularly in circuit architecture. First, transformers are feedforward circuits that receive inputs all at once, whereas the neocortex operates as a recurrent circuit that receives inputs sequentially. Second, the attention module of a transformer is not built from neuron-like units such as ReLU(Wx+b), which are commonly used in standard artificial neural networks. However, these differences may not be fundamental, as RNN-style transformers have been developed and are achieving comparable performance.^15^^,^^157^ Nonetheless, transformers still do not recapitulate neocortical patterns of inter-neuronal connectivity (nor do CNNs). Accordingly, a more rigorous comparison with the neocortex requires consideration of RNN-based circuits with more brain-like connectivity alongside transformers and CNNs.

### Hierarchical attention-based processing as the core of neocortical processing

Focusing on attention-based processing in more detail, unlike transformers, where each layer contains an attention mechanism, traditional neuroscience has implicitly assumed that attentional selection occurs only once during hierarchical processing in the neocortex, and has therefore sought to identify this putative unique site. For example, there has been controversy over whether attention-based signal selection is performed at early or late stages of sensory processing.^158^^,^^159^^,^^160^^,^^161^^,^^162^ To resolve this debate, it has been proposed that the selection stage can be adjusted depending on task demands.^163^ In addition, experiments have supported another theory—the network theory of attention—in which the oculomotor network, responsible for directing gaze to a particular direction, also plays an essential role in directing attention to that same direction.^164^^,^^165^^,^^166^^,^^167^ However, to date, there is no definitive evidence supporting the assumption that attentional selection occurs only once in the neocortex, and the site of attentional selection has remained highly elusive.

By contrast, in transformers, attention-based processing is performed at each layer. This raises an intriguing possibility: if the neocortex similarly performs attentional selection at each hierarchical level, it would naturally explain why the site of attention varies with task demands. Indeed, in human electrocorticography and fMRI studies, signals in higher-order language areas are best explained by the self-attention signals of deep-layered transformers, suggesting, at least at the level of signal correlation, that the neocortex implements hierarchical attentional processing analogous to that of deep-layered transformers.^130^^,^^168^ Furthermore, deep-layered transformers are sufficient to achieve or even surpass human-level performance in diverse tasks, including language processing,^28^^,^^150^^,^^153^^,^^154^ suggesting that hierarchical attention-based processing alone is sufficient to serve as the core computation underlying diverse neocortical functions. Together, we propose that attention-based processing, traditionally regarded as just one of many neocortical functions, is hierarchically organized as a core computational principle underlying diverse neocortical functions, and that in this respect, the neocortex is analogous to hierarchical transformer circuits.

### Cerebellar language processing as a three-layer RNN

Do the similarities observed between the neocortex and AI also apply to the cerebellum? Although the cerebellum is often considered a center of motor control, it is also involved in a wide range of cognitive functions, including language processing.^45^^,^^103^^,^^106^^,^^169^^,^^170^^,^^171^^,^^172^^,^^173^ The right lateral cerebellum (Crus I/II) is essential for two important non-motor language-processing functions: next-word prediction^174^^,^^175^^,^^176^^,^^177^ and grammatical processing, particularly syntactic processing.^173^^,^^178^^,^^179^^,^^180^^,^^181^

**Figure 3:**
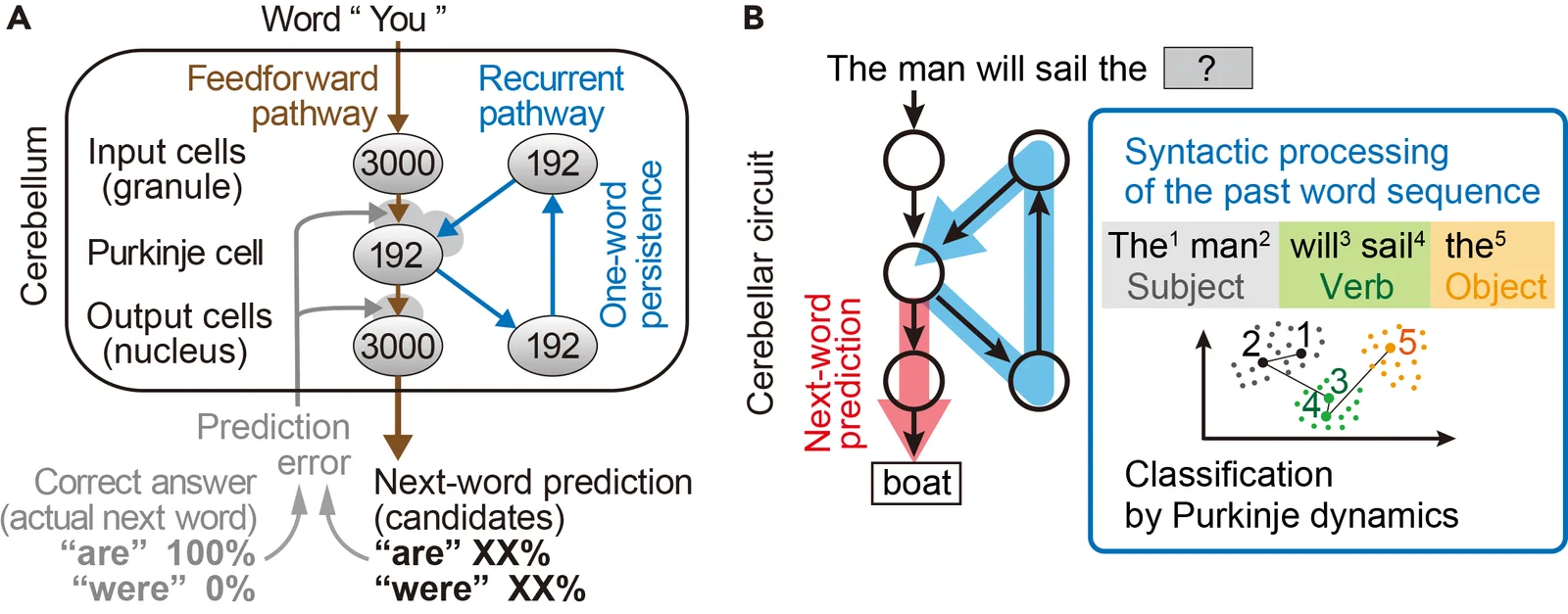
Reproduction of cerebellar language functions by a three-layer artificial RNN imitating the cerebellum (A) A circuit was trained in a cerebellar language function—predicting the next word from a sequence of past words—through prediction-error learning. Each of the 3,000 input neurons (granule cells) represents one word in the 3,000-word set (sparse coding). Purkinje cells integrate the current word information in the feedforward pathway (brown) with the previous word-sequence information in the recurrent pathway (blue). The firing rates of the output neurons (cerebellar nucleus neurons) represent the probabilities that each of the 3,000 words is the next word, forming the next-word prediction. The cerebellum receives the prediction-error signal from the inferior olive through the climbing fiber pathway (gray). This dedicated learning-signal pathway is a feature of the cerebellum, distinct from the neocortex. (B) When the output neurons were successfully trained to predict the next word (red arrow), a circuit for syntactic processing (specifically, classification of words as subject, verb, or object) spontaneously emerged (blue arrow). Panels adapted from Ohmae and Ohmae (2024) (CC BY 4.0).

In cerebellar language processing, the three elements of circuit computation are characterized as follows. In terms of circuit architecture, despite the traditional view of the cerebellum as a feedforward circuit, the output neurons of the cerebellar nuclei (Figure 3A) send extensive feedback projections to the input neurons (Figure S3) via both direct^182^^,^^183^^,^^184^^,^^185^ and indirect projections.^186^^,^^187^^,^^188^^,^^189^ These feedback projections are essential for the predictive functions of the cerebellum.^185^^,^^190^^,^^191^ Considering this recurrent connectivity, the cerebellum can be approximated as a three-layer recurrent circuit. Regarding the input/output and learning, cerebellar internal model theory^23^^,^^33^^,^^35^^,^^36^^,^^37^^,^^38^^,^^39^^,^^40^^,^^41^ has already been extended to the language domain, particularly to next-word prediction (Figure 3A).^106^^,^^174^^,^^175^^,^^192^ On this basis, we constructed a cerebellum-imitating artificial neural circuit and trained it on next-word prediction. Interestingly, the circuit not only acquired the ability to predict the next word (Figure 3B, red arrow) but also spontaneously acquired another cerebellar language function, syntactic processing, in its intermediate layer corresponding to Purkinje cells (Figure 3B, blue).^193^ This was the first brain-imitating artificial circuit to both align with all three elements of circuit computation and reproduce sophisticated cognitive functions characteristic of humans. From the perspective of the world model, this implies that although traditional cerebellar internal model theory has primarily emphasized the cerebellar role as a predictor, the internal model also supports comprehension by abstracting linguistic structures and rules (Figure 1C), thereby revealing parallels with the neocortex and AI.

## Motor language processing

Motor language processing, that is, language generation, involves a multi-step process of forming an idea to be conveyed, planning a sentence by selecting an appropriate combination of words and grammatical structures, and then producing the resulting word sequence primarily through vocalization. Here, we focus particularly on the sentence-planning stage, as it represents a characteristically human form of cognitive processing.

### Shared computational principles of language generation in the brain and AI

Traditionally, the motor language-processing areas (e.g., Broca’s area) have been considered distinct from the sensory language-processing areas (e.g., Wernicke’s area). However, both sensory and motor language processing are now thought to be performed in significantly overlapping regions, including Broca’s and Wernicke’s areas in the neocortex and the right lateral cerebellum.^127^^,^^131^^,^^132^^,^^133^^,^^174^^,^^175^^,^^179^^,^^194^^,^^195^^,^^196^

In language AI, GNMT and BERT have distinct sensory and motor processing units (corresponding to the encoder and decoder units), which aligns with the traditional understanding of language in the brain. In GPT, by contrast, the circuits for sensory and motor processing overlap substantially. GPT also has an encoder-decoder structure, but these are integrated without a clear boundary. During training, GPT generates predictions (Figure 2B, top left) while establishing sentence comprehension. After training, this output system is repurposed for sentence generation by successively selecting the next word (Figure 2B, top right). This overlap between sensory and motor processing in GPT aligns with the more recent view of brain organization.

**Figure 4:**
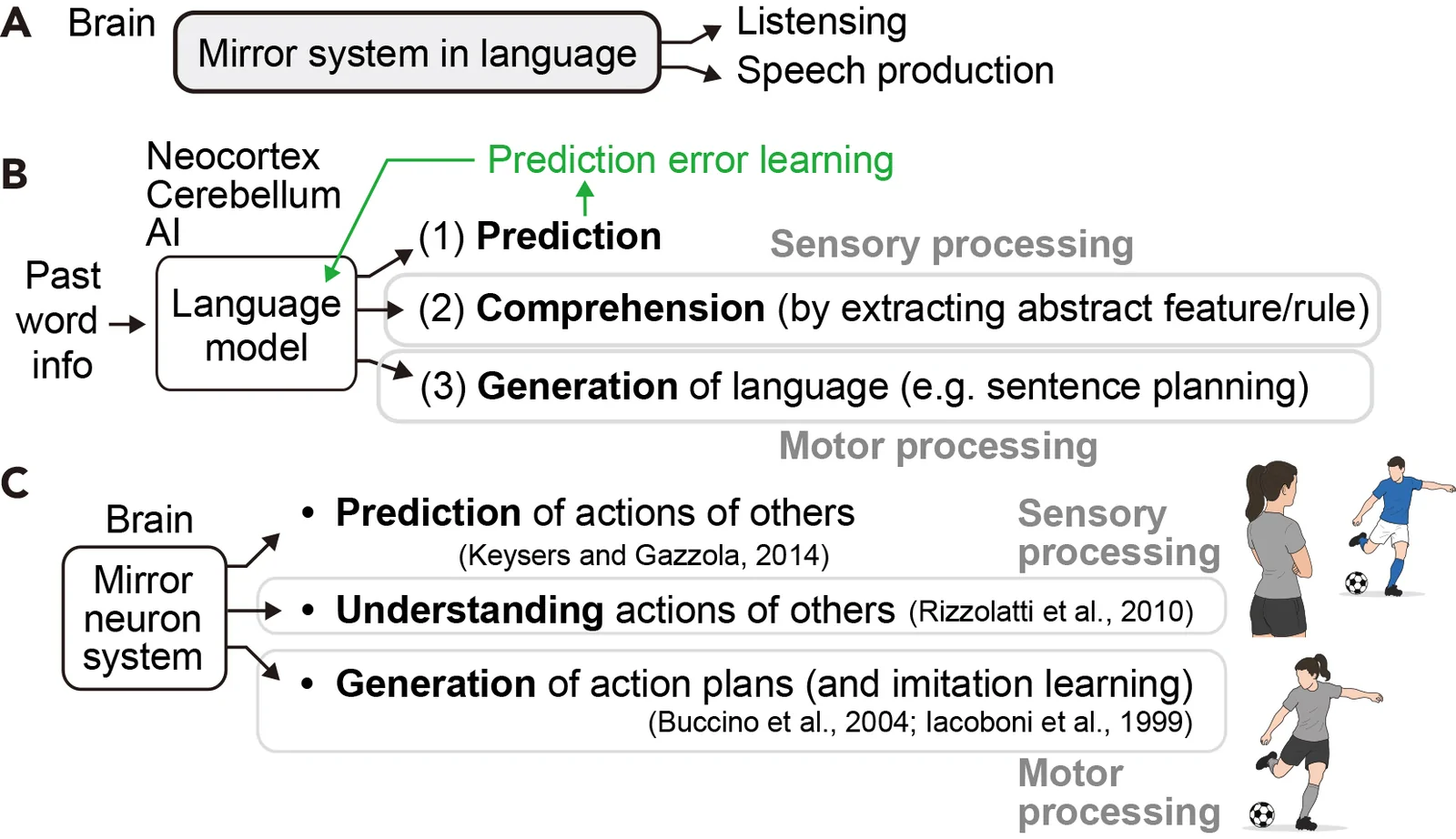
World-model-based processing systems for prediction, comprehension, and generation shared by the brain and AI (A) Mirror neuron theory in language processing. The listening circuit, responsible for perceptual and predictive processing of language, is also crucially involved in language generation. (B) Both the brain (neocortex and cerebellum) and AI acquire language models through next-word prediction learning, thereby establishing a unified processing system for prediction, comprehension, and generation. (C) The mirror neuron system in the brain can be viewed as a world model of actions. Acquired through prediction-error learning, it functions as a unified processing system that (1) predicts the actions of others, (2) observes and interprets the actions of others, and (3) generates action plans.

This raises an important question: does the brain similarly repurpose its word-prediction circuitry for sentence generation? Neuroscience has long proposed that the mirror neuron system—originally developed to explain how circuits for observing and predicting others’ actions are recruited for planning one’s own actions (see below)—can be extended to language, such that circuits for language perception and prediction play a central role in language generation (Figure 4A).^197^^,^^198^^,^^199^^,^^200^ Since 2024, empirical support for this view has begun to accumulate.^201^^,^^202^^,^^203^ In particular, Nishimoto and colleagues demonstrated bidirectional transfer of GPT-based encoder weights between language comprehension and speech generation, indicating shared representations across these processes.^204^ Consistent with this, human single-neuron recordings have identified prefrontal neurons involved in both listening and speaking.^205^ Although traditionally the mirror neuron concept has not been linked to internal model function, it can be reframed as a biological mechanism for reusing predictive models for both comprehension and generation, paralleling autoregressive generation in LLMs, such as GPT (Figure 4B).

In the cerebellum, as described above, the right lateral cerebellum is involved in linguistic prediction and comprehension^193^ and also in planning for sentence generation.^173^^,^^206^ Therefore, although cerebellar function has not traditionally been thought to involve a mirror neuron system, the cerebellum may also implement such a system.

The discussion above reveals striking convergence among autoregressive AI, the neocortex, and the cerebellum: models acquired through next-word prediction can be repurposed to support three seemingly distinct functions: (1) word prediction, (2) language comprehension (representation), and (3) language generation (Figure 4B).

### World models for actions

This parallel, observed in the language domain, also applies to the motor domain. The mirror neuron system, primarily in the higher association areas of the neocortex, is acquired through the prediction of forthcoming actions of others.^207^ Once established, this system engages in both sensory processing, including the interpretation and understanding of others’ actions, and motor processing, including action planning and imitation learning.^208^^,^^209^^,^^210^^,^^211^ This interpretive capacity extends even to abstract representations of others’ mental states and to the inference of the intentions underlying their actions, i.e., theory of mind.^212^ Reframed from the perspective of the world model, the mirror neuron system can be regarded as a mechanism that uses an action model acquired through predicting others’ actions to infer the meaning and intentions of those actions and to apply the same model to the generation of one’s own actions (Figure 4C).

In AI, LLMs have the ability to understand the actions and minds of others, and research repurposing this ability to generate action plans (particularly for robot control) has expanded rapidly since the release of ChatGPT.^213^ A representative example is Google’s PaLM-SayCan, in which PaLM (Pathways Language Model) proposes candidate action sequences in text format and SayCan evaluates and executes the most feasible next action.^214^ For such action planning, video-generative AI (e.g., Stable Diffusion or DALL-E2) can provide candidate action sequences in video format (UniSim). Multiple methods employing video-generative AI for robot control have been proposed, both for direct action planning (UniPi; Google, MIT; RFM-1; GR-1) and for imitation learning to train robot control (TRI).

What these AI approaches have in common is that they use predictive world models to understand the actions of others and then repurpose the same system to generate their own actions, and this structure closely parallels the mirror neuron system in the neocortex (Figure 4C).

### Human-like adaptive language generation through in-context learning

Humans possess a remarkable ability: given appropriate instructions (whether in words, diagrams, or both), they can adapt immediately to novel tasks without additional training—a capacity rarely observed in other animals.^215^^,^^216^^,^^217^^,^^218^ In the context of language generation, such tasks include translation, information provision, product-comment scoring, and other instruction-defined tasks. Recent language AI systems have also achieved a comparable capacity for adaptive responding, widely recognized after the public release of ChatGPT.^151^^,^^219^^,^^220^ Specifically, by receiving instructions known as prompts, GPTs can adapt to a wide variety of new language tasks. This phenomenon, in which the input-output transformation changes in response to the context provided by a given prompt, is called “in-context learning.”^219^^,^^221^^,^^222^ Although the synaptic-weight parameters of the GPT circuit are updated only during training and remain unchanged when adapting to new tasks, the attention mechanism of the transformer retains the prompt-derived contextual signal and continuously propagates this signal throughout the circuit to change the input-output transformation—as if the parameters had been re-optimized in response to that prompt.^221^^,^^223^ The emergence of in-context learning in GPT-3, achieved solely through next-word prediction, was unanticipated even by its developers. Recent analysis revealed that three factors are critical for the acquisition of in-context learning: prediction-error learning, the attentional computation of the transformer, and the large scale of the model.^224^^,^^225^^,^^226^^,^^227^^,^^228^

Although it has been suggested that GPT’s in-context learning and human flexible adaptive capabilities share a common computational principle,^229^ significant architectural differences exist between them. GPT lacks an explicit modular structure; consequently, there are no specific modules or layers that are particularly critical for in-context learning, and its acquisition is attributed to large-scale training of a large attention-based circuit (Figure S4).^151^^,^^219^ In contrast, in the human brain, the lateral prefrontal neocortex plays an essential role in this adaptive capability, and this region is proposed to serve as a hub that selects and switches between specialized neocortical “expert” regions (modules), each having mastered specific tasks, thereby enabling the rapid handling of diverse tasks.^102^^,^^217^^,^^218^^,^^230^^,^^231^ Intriguingly, recent evidence suggests that GPT also employs a form of expert specialization: distinct subcircuits function as hidden expert modules that are dynamically recruited for in-context learning by a multi-head self-attention mechanism.^232^^,^^233^ Moreover, newer LLMs have explicitly incorporated the mixture-of-experts architecture—which, like the neocortex, selects and switches between specialized modules—into their transformer design (e.g., DeepSeek), and these LLMs achieve in-context learning performance comparable to GPT while using substantially smaller circuits.^234^^,^^235^ Although empirical evidence for this similarity is still limited, these findings suggest that the combination of attention mechanisms and mixture of experts constitutes a shared computational principle for in-context learning across both biological and artificial intelligence.

### Convergent evolution of language AI and the human brain

The evolution of language AI shows an intriguing convergence with human language processing, primarily at the algorithmic level. Initially, GNMT relied on supervised learning using human-translated texts. BERT then advanced to unsupervised learning (via masked-word completion), thereby acquiring a more general-purpose capacity for language comprehension. GPT further pushed this evolution by adopting next-word prediction, which enabled sentence generation in a way that masked-word completion could not readily support. This generative strategy is referred to as autoregressive generation. Interestingly, this evolution in language AI was not inspired by neuroscientific theories of language processing, but rather has gone on to influence neuroscience research.^130^^,^^138^^,^^139^^,^^140^^,^^142^

Furthermore, as a macro-level convergence, we propose that the neocortex, cerebellum, and autoregressive generative AI share a form of world-model-based computation that integrates prediction, comprehension, and generation (Figure 4B). Next-word prediction is well suited for acquiring such integrated systems, and training data for prediction are naturally abundant.^28^ It is therefore likely that the brain and AI independently converged on this strategy. Moreover, in AI, attention mechanisms have enabled broad adaptability to novel tasks, and the mixture-of-experts mechanism has improved the efficiency of this acquisition, suggesting a further algorithmic-level parallel with the neural basis of human intellectual flexibility. Taken together, this multi-scale convergence suggests that the brain and AI share the computational principles underlying human-level intelligence, and that a deeper understanding of these principles will be central to future efforts to bridge neuroscience and AI.

## Discussion

### Toward a new neuroscience theory of diverse functions and intelligence

Drawing primarily on comparisons among neocortical predictive coding, cerebellar internal-model processing, neocortical mirror-neuron-system processing, and autoregressive generative-model processing in AI, this paper offers the following insights into the computational principles of the brain. The neocortex and cerebellum predict future states of the external world from past inputs and learn by minimizing prediction error. Through this process, they compress and abstract information about the external world to form compact yet powerful world models. They then repurpose the predictive outputs of these models for motor output generation. We propose that this world-model-based computation constitutes a core principle through which the neocortex and cerebellum achieve diverse functions despite their relatively uniform circuit structures. In addition, in contrast to the traditional neuroscientific view of attention as just one function among many, we propose that hierarchically organized attention-based processing in the neocortex constitutes a core computational basis for the cognitive functions characteristic of humans, and that in this respect, the neocortex resembles hierarchical transformer circuits. Finally, we propose that attention-based switching among expert modules forms a foundation for advanced intelligence, particularly the immediate adaptability to novel tasks observed both in the neocortex and transformer-based AI.

Future evidence supporting this hypothesis—that predictive world models are reused for sensory understanding as well as motor and language generation—would include evidence of shared or partially overlapping circuits and internal representations across prediction, understanding, and generation, as well as evidence that interventions in prediction-related circuits also affect understanding and generation. Conversely, if future studies show that these three functions rely on fundamentally independent circuits and representations, the validity of this hypothesis would be weakened. A deeper understanding of world-model-based computation will be essential not only for advancing neuroscience and AI separately but also for bridging the two fields and elucidating the core principles of intelligence.

### Predictive world models as a foundation for higher cognitive functions

How does world-model-based computation contribute to advanced intelligence in humans and AI? We address this question by focusing on reasoning abilities grounded in causal understanding. We propose that predictive world-model-based processes provide a necessary, though not necessarily sufficient, foundation for the development of causal and counterfactual reasoning.

In AI, large language models trained solely through prediction-error learning already exhibit substantial multi-step reasoning, some capacity for causal judgment, and counterfactual reasoning, at least in simple tasks.^236^^,^^237^^,^^238^^,^^239^^,^^240^^,^^241^^,^^242^^,^^243^ Furthermore, these abilities can be significantly enhanced through reinforcement learning, in which criteria for sound reasoning and procedures for counterfactual reasoning are trained more explicitly. Nevertheless, reinforcement learning appears to elicit and refine skills already acquired through prediction-error learning, rather than create entirely new abilities from scratch.^241^^,^^242^^,^^244^ This suggests that predictive world models, even if not a sufficient condition for higher-order reasoning, play an indispensable foundational role.

In humans, much of early cognitive development—prior to formal education—proceeds through unsupervised and implicit learning.^245^^,^^246^^,^^247^ Prediction-error learning plays a critical role in this process, driving the formation and updating of internal models.^4^^,^^7^^,^^246^^,^^247^^,^^248^^,^^249^^,^^250^ Foundations of causal reasoning are already evident by around 2 years of age, and precursor forms of counterfactual reasoning emerge by around 6 years.^251^^,^^252^^,^^253^ These early abilities are thought to provide a foundation for the more abstract causal understanding seen in adults.^254^ Taken together, these findings suggest that internal models formed through prediction-error learning in early development, while not sufficient for adult-level reasoning, constitute an important and probably indispensable foundation. However, because humans cannot be observed under conditions in which internal models are formed solely through prediction-error learning, the contributions of other forms of unsupervised learning, supervised learning, and innate mechanisms must also be considered.

### World-model-based computation in the brain

The view that the neocortex and cerebellum implement diverse functions through world-model-based computation can provide an important interpretive framework for future neuroscience research. For example, reward signals in these regions have been interpreted as learning signals for improving behavior in model-free reinforcement learning.^43^^,^^45^^,^^255^^,^^256^^,^^257^^,^^258^^,^^259^^,^^260^^,^^261^^,^^262^^,^^263^ This is because animal behavior in psychological tasks—where animals can fully sample a limited set of task states—can be explained by classical model-free reinforcement learning that improves action selection based solely on the value of previously experienced actions and states.^255^^,^^256^^,^^257^^,^^258^^,^^259^^,^^260^^,^^261^^,^^262^^,^^263^ However, the human brain also implements model-based reinforcement learning: it uses world models in the neocortex and cerebellum to simulate unexperienced situations^54^^,^^56^^,^^264^^,^^265^ and determines action selection based on the inferred values of novel actions and states.^54^^,^^266^^,^^267^^,^^268^^,^^269^ From the perspective of world models, a reward is simply one type of event to be predicted, alongside other environmental events. Reward-related signals can therefore be interpreted either as prediction signals of that event or as signals related to the prediction-error learning of that event (e.g., ground-truth signals and error signals). Taken together, these considerations suggest that the reward signal in the brain is not necessarily limited to being the learning signal for improving behavior. In future research, the world-model perspective will lead to more careful and accurate interpretation of neural signals.

### Implications of viewing the neocortex as a hierarchical attention circuit

Viewing the neocortex as a hierarchical attention-based processing circuit has the potential to reshape future research on neocortical computations, which have traditionally been described as a collection of numerous distinct processes. For example, the parietal neocortex is typically described in terms of two distinct roles: attention and movement intention.^270^^,^^271^ However, recent advances in transformer circuits, where attention and intention are integrated into a single circuit computation, suggest that these functions may represent different aspects of a unified process. Similarly, the prefrontal neocortex is currently associated with more than ten distinct functions^272^; yet given that attention-based processing in transformers can flexibly achieve apparently diverse functions through in-context learning, the fundamental processing in the prefrontal neocortex may be better understood as attention-based computation at higher hierarchical layers. Such reinterpretation of diverse neocortical functions within a framework of hierarchical attention-based computation, together with comparison to transformer computation, can substantially advance the field of neuroscience.

### Leveraging AI insights to understand neurological disorders

Insights from AI research can deepen our understanding not only of the brain but also of neurological disorders. One of the major mysteries of neurodevelopmental disorders is that their onset is probabilistic and not always reproducible. For instance, autism involves hundreds of genetic and non-genetic risk factors, with the interaction of multiple risk factors considered critical.^273^^,^^274^ However, no decisive combination of interactions guarantees onset. By analogy with the fact that learning in AI circuits is also probabilistic, neurodevelopmental disorders may be viewed as probabilistic impairments in learning. In AI development, reducing the circuit size, improperly designing the circuit architecture, and choosing an unsuitable learning method destabilize the learning process and reduce the success rate. From this perspective, we infer that in neurodevelopmental disorders, genetic and non-genetic risk factors destabilize learning and can result in the failure to achieve the appropriate developmental trajectory, ultimately leading to the onset of a disorder. By creating brain-imitating circuits and introducing manipulations to simulate these risk factors, we may significantly advance our understanding of the mechanisms underlying the onset of neurodevelopmental disorders.

### Scaling and the emergence of intelligence

The key factor behind recent advances in AI was the scaling up of simple circuits equipped with attention mechanisms alongside massive increases in training data.^275^^,^^276^^,^^277^ The conventional belief was that excessive scaling of circuits results in too much capacity, leading to overfitting and reduced generalization to new data. However, in practice, large-scale AI circuits did not lead to overfitting. Instead, scaling up transformer circuits led to the emergence of in-context learning. A representative hypothesis for the underlying mechanism is the “lottery ticket” hypothesis (Figure S5),^278^^,^^279^ which proposes that (1) increasing the size of a circuit significantly raises the probability that it contains a subnetwork with complex processing capabilities prior to learning, and (2) after learning, only a small fraction of the circuit actually contributes to the output. Interestingly, corresponding observations have been reported in the human brain, with its vast number of synapses (∼100 trillion). For point (1), there is already considerable similarity between the language signals in an untrained transformer circuit and those in the neocortex.^139^ For point (2), weak synapses are pruned, and the associated molecular mechanisms have been identified (e.g., Arc’s tagging of weak synapses).^280^ These parallels suggest that circuit scale expansion itself has contributed to the human brain’s exceptional flexibility and intelligence relative to other species. The perspective that intelligence resides not in elegantly designed circuits but in large-scale attention circuits will be pivotal in advancing neuroscience.

Nevertheless, the human brain learns far more rapidly than LLMs from vastly fewer examples and exhibits superior computational efficiency; indeed, the number of words required for humans has been estimated to be roughly one ten-thousandth that required to train AI systems. As noted above, newer-generation LLMs have improved learning and computational efficiency by incorporating brain-like mixture-of-experts mechanisms, yet they still fall far short of the brain’s efficiency. This gap may reflect the brain’s implementation-level circuit organization. Transformers consist of almost entirely homogeneous circuit structures, whereas both the neocortex^281^^,^^282^^,^^283^ and cerebellum^284^^,^^285^ exhibit fine-grained cellular heterogeneity and microcircuit specialization that can contribute to distinct functions. Such heterogeneity and specialization can substantially contribute to the efficient learning of diverse functions from limited data.^286^^,^^287^ Thus, in light of these remaining advantages of the brain, AI will continue to draw inspiration from the brain, while neuroscience will continue to gain new insights from AI.

### Beyond convergence: Illuminating intelligence through reciprocal development between neuroscience and AI

This paper integrates previously scattered observations of brain-AI similarities across multiple functional domains by examining their computational principles through the lens of the world model, a concept common to both. In doing so, we identified two features shared across AI, the neocortex, and the cerebellum: (1) prediction-error learning and (2) the integrated processing of prediction, comprehension, and generation. We further identified two features shared specifically by transformer-based AI and the neocortex: (3) hierarchical attention-based processing and (4) adaptability to novel tasks via attention-based processing and mixture of experts. Together, these four parallels define a set of algorithm-level correspondences between the brain and AI.

Although our analysis focused on the neocortex and cerebellum, extending this comparative approach to other brain regions and functions—such as reinforcement learning in the brain and AI—promises to deepen our understanding of these functions and reveal new insights into underlying computational mechanisms. Importantly, the identified convergence points offer actionable guidelines for AI design: aligning even more closely with the brain’s computational strategies at these convergence points may enable more efficient AI systems, while such brain-inspired AI will, in turn, substantially advance our understanding of human intelligence. The deepening dialog between neuroscience and AI promises to benefit both fields, ultimately guiding us toward a fundamental understanding of intelligence itself.

## Acknowledgments

This work was supported by the Beijing Key Laboratory of Brain Science and Brain-Machine Interface. The authors thank lab member Chun Zhao for assistance with figures and references and former lab members Tuo Xin, Ouni Cao, and Zoha Hassan for their contributions to this work. We are sincerely grateful to Professors Tatsuo Okubo (CIBR), Joji Tsunada (CIBR), Akihiro Yamanaka (CIBR), Tadahiro Taniguchi (Kyoto University), and Takahiro Shinozaki (Science Tokyo) for their invaluable insights and constructive comments, which greatly strengthened the manuscript. Finally, we thank Dr. Lindsay Bremner for professional editing assistance.

## Author contributions

S.O. conceived the study and prepared the initial manuscript and figures, incorporating substantial input from K.O. Both authors jointly revised the text and figures and contributed to the finalization of the manuscript.

## Declaration of interests

The authors declare no competing interests.

## Declaration of generative AI and AI-assisted technologies in the writing process

During the preparation of this work, the authors used ChatGPT in order to support stylistic consistency and the paraphrasing of selected passages. After using this tool, the authors thoroughly reviewed and edited the content and take full responsibility for the content of this publication.

## Supplemental information

Document S1. Figures S1–S5

Document S2. Article plus supplemental information
