# Supplementary material for: Brain-AI convergence: Generative world models and hierarchical attention for human intelligence
Source: Patterns (N Y). 2026 Jun 26;7(8):101593. doi: 10.1016/j.patter.2026.101593 (PMC13494634; doi:10.1016/j.patter.2026.101593)
Supplement: Supplementary file 1 [file mmc1.pdf]

Patterns, Volume *7*

Supplemental information

Brain-AI convergence: Generative world models and hierarchical attention for human intelligence

Shogo Ohmae and Keiko Ohmae

Supplementary Figures

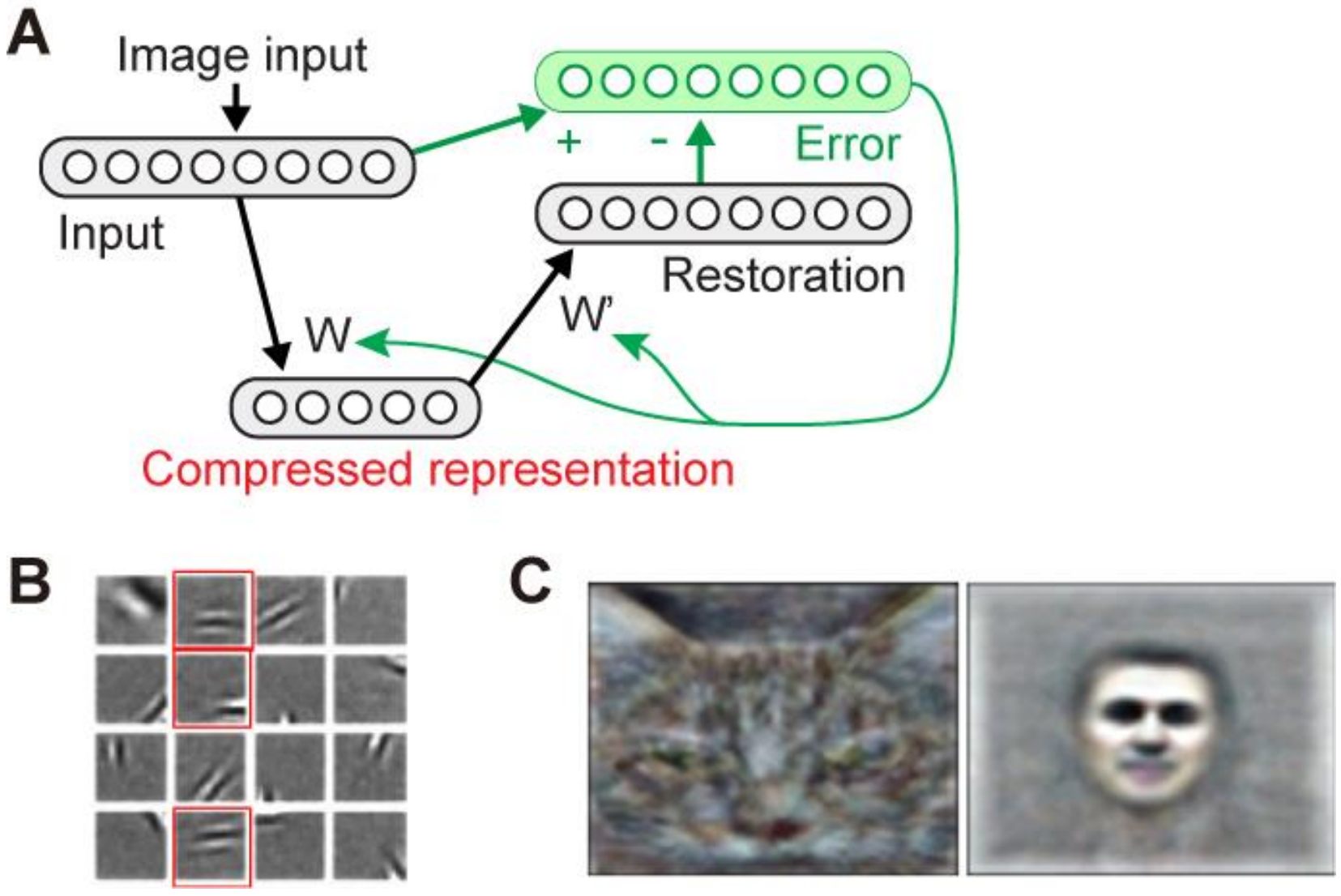

Figure S1. Deep-layered autoencoders as theories of visual information processing in the neocortex. A, Autoencoder circuit. In a representative autoencoder circuit, the input neurons receive a 2D visual image (e.g., the pixel information of a photograph). The restoration neurons (downstream via disynaptic projections) are trained to encode the same information as the input neurons. If the downstream after the input neurons has fewer neurons, the input information needs to be compressed into a more compact dimensional space (red; compressed representation neurons). The error neurons calculate the difference between the input neurons and the restoration neurons, and the synaptic weights (W and W') of the circuit are updated to minimize this error. [Autoencoder] Circuit structure: This representative autoencoder circuit is a classical three-layer neural network (although various types of circuits can be trained to acquire the autoencoder function). Input/output: The input is a 2D visual image, and the output is the image reconstructed by the restoration neurons. Additionally, the signals from the compressed-representation neurons (red) serve as a secondary but more processed output (i.e., a de facto output). Learning: The synaptic weights (W and W') are updated to minimize the reconstruction error through unsupervised learning. The error signal (generated by the error neurons) is used solely for learning (green) and does not contribute to the outputs of the information processing. **B,** Neurons in the autoencoder circuit in **a** acquired receptive fields with local line/stripe patterns (described by Gabor functions, suitable for object contour/edge detection), as in the primary visual neocortex. The red frames highlight neurons with receptive fields that prefer horizontal lines/stripes in different local areas of the visual field. Furthermore, a phenomenon was observed in which the majority of receptive fields changed according to the characteristics of the training data, similar to the phenomenon in which vertical stripe receptive fields predominate in the visual neocortex of cats raised in a vertical stripe environment (Blakemore and Cooper, 1970). **C**, The optimal stimuli that most strongly activate two representative neurons in the highest layer of the deep-layered (hierarchical) autoencoder (Le et al., 2012). Left: The best activation stimulus for one neuron was this cat-like feature (Google's cat); this neuron is interpreted as a cat-classification neuron. Right: A human-face-classification neuron, whose best stimulus was a human facial feature, also emerged. [Deep-layered autoencoder] Circuit structure: A deep circuit consisting of three repetitions of the receptive-field learning sub-layer (**a**) combined with a pooling sub-layer and a contrast-normalization sub-layer. Input/output: A deep-layered structure where the signal compressed in a lower layer is used as the input for the next layer. Learning: The three receptive field sub-layers are trained sequentially, one layer at a time, starting from the lowest layer, to reconstruct the input signal (greedy layer-wise training). Panel **c** adapted with permission

from Le et al. (2012). Panel **B** adapted from Olshausen & Field (1996) with permission from Springer Nature. Panel **C** adapted with permission from Le et al. (2012).

Deep-layered autoencoders as a circuit computation theory of the visual neocortex

To acquire models of the external world and understand the world, the brain generally needs to learn information processing with little instruction on the correct processing results [1,2]. The visual recognition circuit, located in the cerebral visual neocortex, is primarily trained through unsupervised learning [1-6]. In learning theory of the neocortex, unsupervised "autoencoder" learning is essential [2-4]. In autoencoder learning, the original input image serves as the target information to learn (**Figure S1A**). In the autoencoder circuit, the input neurons receive a 2D visual image and the circuit is trained so that the restoration neurons restore exactly the same information as the input, i.e., the error between the two populations is minimized. It is called the autoencoder in machine learning terms, because it is trained to encode (ouput) the input itself. When the downstream neurons (red) is fewer then the input neurons, the input information needs to be compressed into a more compact dimensional space, resulting in the generation of compressed visual information as a secondary output.

The idea that the visual neocortex can function as an autoencoder has a long history [4]. In fact, when an autoencoder circuit of an artificial neural network (ANN) was trained with numerous natural images, the compressed-representation neurons acquired receptive fields similar to those of the primary visual neocortex (**Figure S1B**) [7], suggesting that the primary visual neocortex also performs autoencoder-like feature extraction.

To acquire more high-level and abstract feature extraction, researchers created a deep-layered (hierarchical) autoencoder by stacking the data-compression functions of autoencoders, but training all layers simultaneously by backpropagation proved challenging. To overcome this challenge, in 2006, Hinton group proposed greedy layer-wise training, in which the deep autoencoder was trained sequentially from lower to upper layers [8,9], analogous to the critical period in the neocortices (early anchoring in lower visual areas) [10]. Using this learning procedure, in 2012, a group of researchers at Google successfully enabled neurons at the highest layer of a deep autoencoder to acquire specific responses to and recognition of human and cat faces (**Figure S1C**). This was achieved by implementing tolerance sub-layers (more concretely, a pooling sub-layer and a contrast normalization sub-layer) in each layer, as first proposed in the "neocognitron" network [11], and by training the circuit through unsupervised autoencoder learning with large amounts of image data [12]. This result demonstrated the remarkable potential of the deep autoencoder to autonomously learn not only feature extraction of visual information but also object recognition and classification.

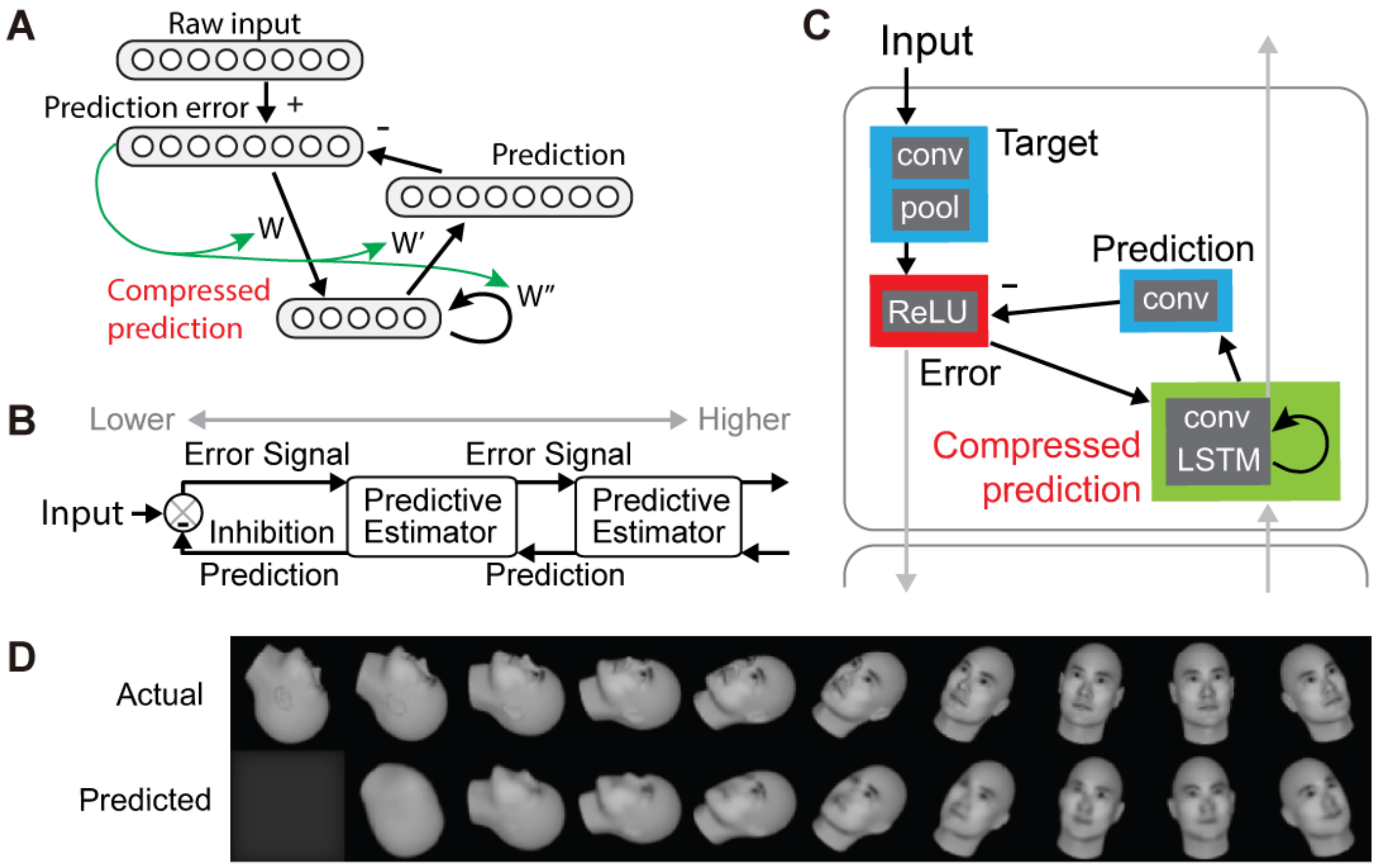

Figure S2. AI circuit similar to the predictive coding circuit, a processing theory for dynamic visual information in the neocortex.

A, Structural diagram of a predictive coding circuit. Raw input information is processed by computing the difference between the input and the prediction, with only the resulting prediction error transmitted downstream. The compressed prediction neurons utilize a recurrent connection to integrate the current prediction-error signal (synaptic weight W) with the previous compressed prediction signal (W'') and transmit a new signal (W') to the prediction neurons. The prediction neurons restore the original data dimensions and predict the next input. The error signal (from the prediction-error neurons) is central not only for learning but also for information flow in the circuit. When the input is a static image, the past and new inputs are identical, and the predictive neurons reconstruct the input; this is equivalent to the autoencoder process. **B**, A schematic diagram of a deep-layered predictive coding circuit. Each layer (corresponding to the entire circuit in **A**) sends a copy of the error signal to the next higher layer and receives a copy of the compressed prediction from that higher layer. Since error signals are generated and consumed within each layer during learning, there is no need for backpropagation to transmit them to distant layers. Circuit: A raw input layer followed by a 3-layer recurrent neural network. Input/output: The input is a 2D visual image. The primary output is the prediction of the next input. With a deep-layered structure, secondary outputs are the prediction-error signal that is sent to the next higher layer and the compressed prediction sent to the lower layer. Learning: The synaptic weights of the circuit are updated to minimize the prediction-error signal (the learning signal is shown in green). **C,** Deep predictive coding network (PredNet). In terms of input/output signals and the learning process, PredNet faithfully implements the neocortical theory of deep predictive coding, incorporating the core circuitry shown in **a** with additional convolution sublayers, a pooling sublayer, and a long short-term memory (LSTM) unit. The LSTM integrates temporal information via a loop pathway (green). The inputs and outputs across layers are depicted with gray arrows. ReLU, rectified linear unit. **D,** Prediction by PredNet of the next frame in a sequence of 2D images (a face rotating in 3D space). PredNet successfully achieved higher prediction accuracy than either a CNN-RNN or an autoencoder tested by the authors.

Panel **B** adapted from Rao & Ballard (1999) with permission from Springer Nature. Panels **C,D** adapted with permission from Lotter W. et al. (2016).

Evolution of circuit computation theory of the visual neocortex: Extension of autoencoders to time-directional information-integration circuits

Deep-layered autoencoders and CNNs have succeeded in reproducing certain theoretical aspects of the circuit computations of the visual neocortex. However, they differ from the brain in their inability to integrate information in the time direction. Deep autoencoders and CNNs are feedforward circuits, and processing limitations of their structure prevent them from integrating past and current information, making them incapable of processing dynamic or sequential visual images (videos) [16,63-74]. By contrast, the neocortex, with multiple recurrent connections, is able to integrate past and present information and process dynamic information that changes over time [70,75-77]. To resolve this mismatch, autoencoder circuits need to be extended to be suitable for integration of information over time. The first candidate circuit structure is the RNN, which integrates information over time via recurrent pathways. (Transformer circuits, despite their feedforward structure, can also integrate information over time by extending the input format; discussed later.) For RNN input/output signals and learning, we propose "prediction-error learning"—where the circuit outputs predictions of future inputs on the basis of past inputs from the external world and learns from the prediction errors—for two reasons. First, predicting the future is equivalent to predicting dynamic changes in the external world; therefore, the circuit can acquire models of these dynamics through prediction-error learning. Second, prediction-error learning allows for numerous cycles of unsupervised learning, similar to the brain, which relies on extensive unsupervised learning [1,22,78-80].

A theoretical circuit that fits these criteria has already been proposed for the neocortex: the predictive coding circuit (**Figure S2A**) [31,81-83]. Predictive coding was originally proposed to explain receptive field properties that could not be explained by classical theories of the visual neocortex. A growing body of experimental evidence supports this theory [8,82,84]. The theory has also been generalized to other sensory areas, including the somatosensory and auditory neocortices [8,85-89]. The predictive coding circuit resembles the autoencoder circuit but differs in that the prediction-error signal is transmitted downstream and that the circuit with a recurrent pathway can receive inputs sequentially to integrate past and current information (**Figure S2A**). When the input is a still image, the processing is equivalent to the autoencoder process, thus the predictive coding circuit can be interpreted as an RNN-oriented extension of the autoencoder, to enable information integration over time.

Predictive coding circuits can be stacked to form a deep-layered structure to incorporate the hierarchical structure of the visual neocortices, with reciprocal connections between the layers [14,82,83,90]. In a deep-layered predictive coding circuit, prediction-error signals that cannot be predicted in a lower layer are sent to the next higher layer to become new prediction targets (**Figure S2B,C**) [31]. This higher-level prediction signal is fed back as a top–down signal to the lower layer. Moreover, the

free-energy principle, which mathematically formulates the prediction error in predictive coding circuits as entropy, focuses on the fact that the dynamics of the world are hierarchical and proposes that the world can be modeled by mapping the dynamics onto a deep-layered predictive coding circuit [12,14].

Prediction-error-learning RNNs as video processing AI: PredNet, CNN-RNN

An AI circuit inspired by deep-layered predictive coding theory in the neocortex is the deep predictive coding network (PredNet) (**Figure S2C**) [75,76]. PredNet is a practical implementation of the circuit theory of the visual neocortex, although its use of long short-term memory (LSTM) circuits for its RNN component does not replicate the circuit structure of the neocortex (LSTM is designed for efficient backpropagation and the effective integration of past and present information through gate functions). PredNet achieved high prediction accuracy for the next frame of rotating objects, such as a human face, in a 3D environment (**Figure S2D**). Furthermore, it developed internal representations that efficiently coded object parameters (e.g., identity, view, rotation speed) in the intermediate layer. This demonstrates that PredNet replicates certain functional aspects of the neocortex that autoencoders could not.

PredNet exemplify RNN circuits, through prediction-error learning, can acquire world models that represent latent information absent in raw sensory data, such as 3D structures, by abstracting the information necessary for prediction. The neocortex also acquires internal models of the external world through experience [12,14,26-37,95] and extracts highly abstract sensory information at higher levels of the cortical hierarchy. This similarity indicates that the neocortex acquires world models through prediction-error learning, utilizing the compressed and abstract information within these models to interpret the external world.

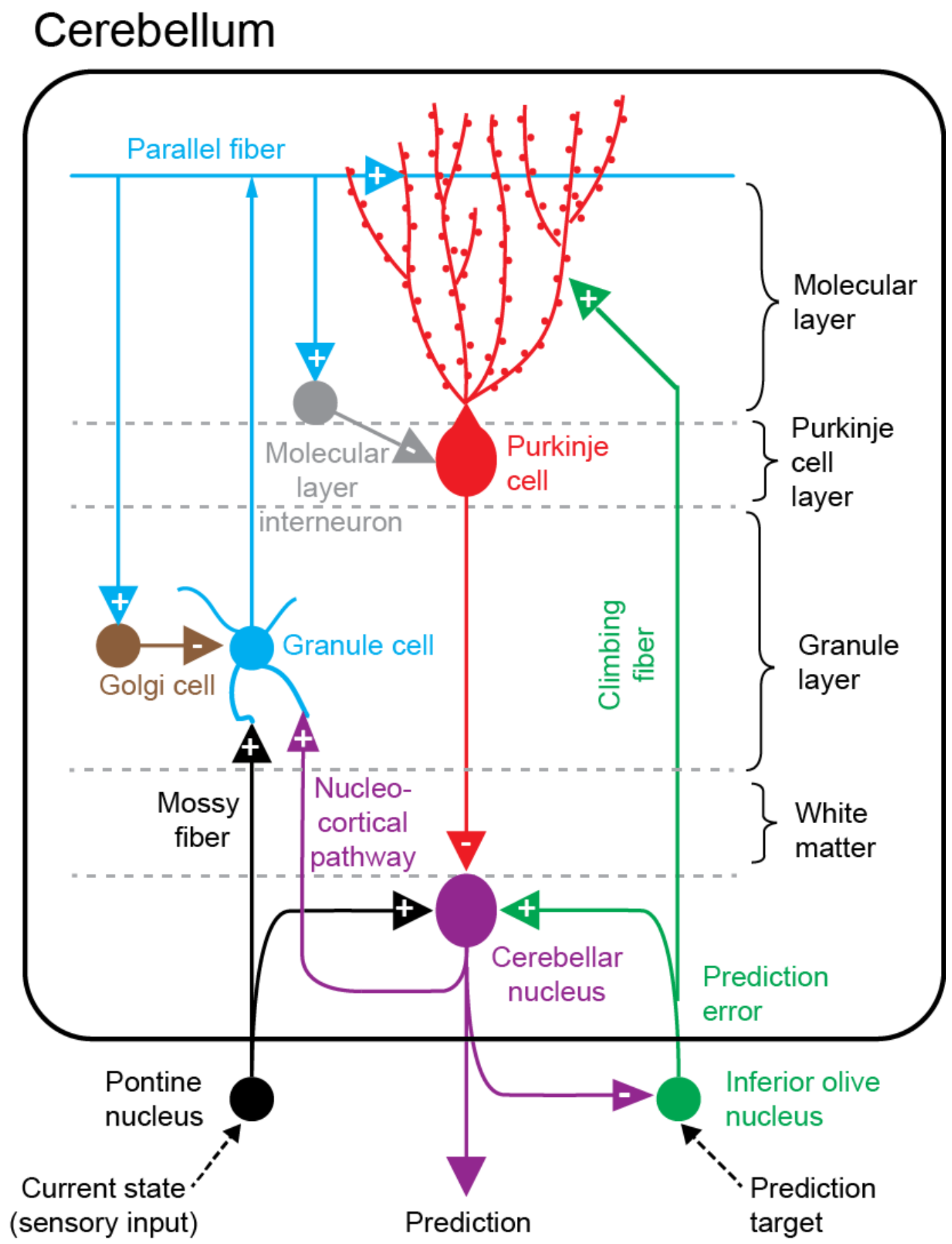

Figure S3. Local cerebellar circuitry. The pontine nucleus sends the signal of the current state of the world to the cerebellum (cerebellar cortex) via mossy fibers. These fibers synapse onto granule cells, whose axons—the parallel fibers—convey this information to Purkinje cells. Purkinje cells and their downstream cerebellar nuclear neurons together generate predictive signals about future states of the world, which constitute the cerebellar output. In the inferior olive, the cerebellar predictive output is compared with actual sensory input (as the target of the prediction), and any discrepancy gives rise to a prediction-error signal. This error signal is relayed back to Purkinje cells and cerebellar nuclear neurons via climbing fibers, where it drives synaptic weight updates that refine the cerebellar predictive accuracy. The principal credit-assignment mechanism underlying these updates is long-term depression (LTD), which selectively weakens parallel fiber–Purkinje cell synapses that were active immediately before the prediction-error input of climbing fibers.

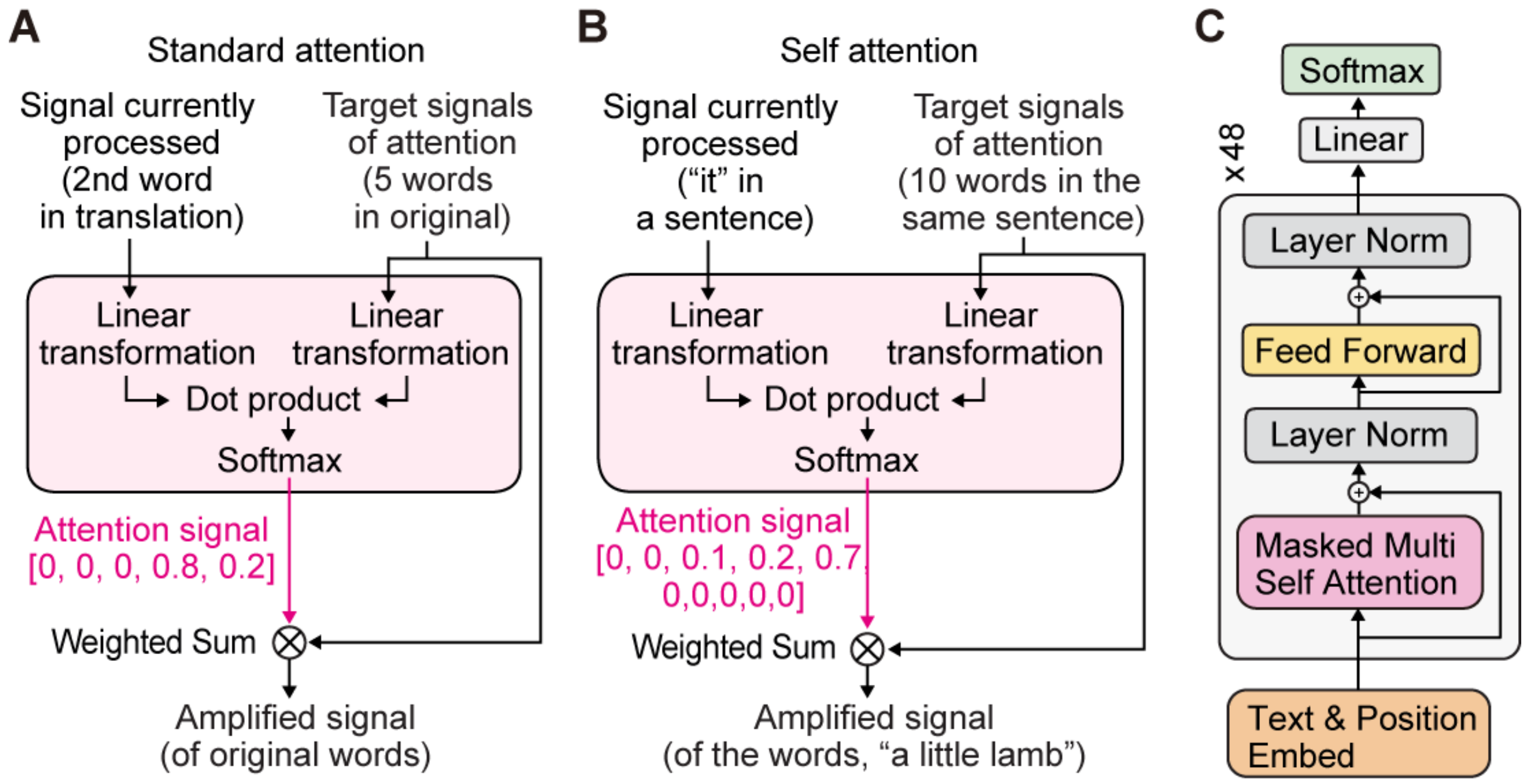

Figure S4. Transformer attention circuit. **A**, Standard attention circuit. For simplicity, this will be explained with a concrete example. Consider the case where, during the translation of a sentence with five words, the signal for the second word that was most recently translated is used when generating the next word (input section). When selecting the next word, assuming that focusing on the corresponding word in the original sentence will lead to a more accurate and faithful translation, the attention mechanism (red box) generates an attention signal (degree of focus) for each word in the original sentence, with the sum being equal to 1 (red arrow; e.g., [0, 0, 0, 0.8, 0.2]). Next, this attention signal is used to weight the signal for each of the five words in the original sentence (with the attention signal serving as the weight coefficient), and the circuit outputs the original word information with the words of interest highlighted (output section). The first input is a high-dimensional signal (e.g., a 512-dimensional signal), which provides the context of the current processing. The second input is the five 512-dimensional signals corresponding to the five original words. In the attention mechanism of transformer predecessors (e.g., GNMT), this input/output was learned in a two-layer neural network (called additive attention; see **Figure 2A**). By contrast, the dot-product attention mechanism in the transformer assumes that this input/output is, in this case, converting the second translated word (the first input) into candidates for the third word, transforming dimensions from the original language to the target language, and then calculating importance by comparing it with each of the five original words (the second input). It also assumes that the conversion to the next word and the dimensional transformation can be done only by linear transformation (rotation and scaling operations in 512 dimensions), and the importance calculation is done via a dot-product operation (similarity calculation) (inside the red box). This dot-product attention mechanism, owing to the rather aggressive assumptions, is much faster in computation and learning than additive attention, yet, surprisingly, maintains comparable accuracy (to be precise, the linear transformation is applied to both input signals; in practice, to achieve higher performance, a multi-head attention process is employed, where eight linear transformations are performed in parallel to obtain eight parallel candidates of the attention signal). **B**, Self-attention mechanism. To understand "it" in a sentence, "Mary had a little lamb, and it was very white", the self-attention mechanism generates an attention signal that indicates which words in the

sentence to attend to. The first and second inputs are signals from the same sentence (e.g., the signal for the word, “it”, and the signal for the ten words of the sentence). The processing steps are the same as in **A**. If the word "it" corresponds to "a little lamb," the self-attention mechanism focuses on these three words and creates a signal to represent "a little lamb" by multiplying the attention coefficients (red arrow) and the word signals of the sentence (i.e., the copy of the second input) so that the output represents the detailed meaning of "it" (the information regarding "it" itself is also conveyed downstream by bypassing the attention mechanism; see the bypass path in **C**). **C**, Transformer circuit of GPT-2. Restatement of **Figure 2B**.

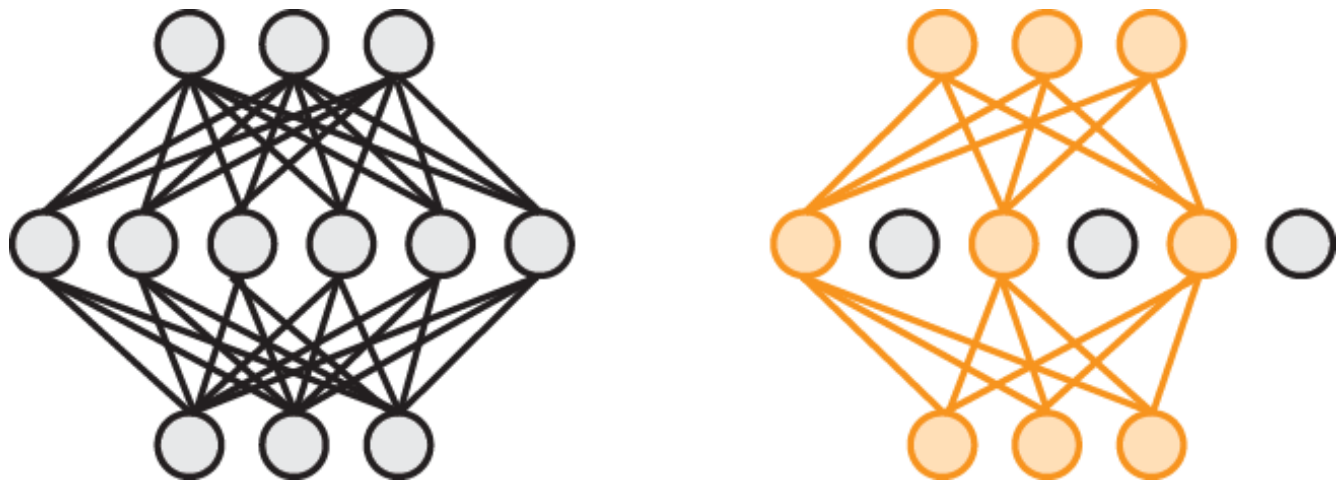

Figure S5. Significance of scaling up the circuit size to intelligence and the “Lottery Ticket Hypothesis”.

A representative hypothesis, known as the "lottery ticket hypothesis," proposes the following mechanism for how circuit scaling contributes to the acquisition of intelligence. First, an artificial neural circuit contains numerous subnetworks, and as the circuit is scaled up, the number of the subnetworks increases exponentially. For example, in a three-layer 3-6-3 network (black), the number of 3-3-3 subnetworks (orange) is Combination(6, 3) = 20. These subnetworks overlap, but no pair has exactly the same initial value combination). During learning, a subnetwork whose initial values happen to be well-suited for task-demanding processing contributes to the output and is reinforced, eventually playing a major role in processing of the entire circuit. Therefore, larger circuits are more likely to contain such a "hit" subnetwork and to be successfully trained. This hypothesis is experimentally supported [31,32].

The lottery ticket hypothesis implies two points: (1) As the circuit scales, the number of subnetworks increases sharply, boosting the likelihood of containing subnetworks with complex processing capabilities. (2) Even if a large circuit is being trained, only a small fraction of the circuit actually contributes to the output generation after learning (This is called “implicit regularization” because it is as if a rule that facilitates learning in only a small fraction of the synapses has been added). Point (1) suggests that when learning sophisticated information processing like in-context learning, which requires complex processing mechanisms (see “Immediate Adaptability to Novel Tasks” in motor language system)[33,34], the existence of a pre-learning subnetwork that happens to be well-suited for such complex processing beforehand is essential for successful learning. In line with (2), it was found that only a small portion of the vast number of synapses in the large AI circuit are used after training, and that even if 90% or more of weak synapses are pruned (i.e., further reduced to zero), most of the performance gained from learning can be maintained [35-38]. This in turn means that a large-scale circuit is necessary only during the learning phase [31,32].

The importance of circuit scale in neuroscience

The fact that large-scale circuitry in AI was a key factor in acquiring advanced processing capabilities had a profound impact on AI research and computational neuroscience, where researchers had been focusing on designing intelligent circuits utilizing sophisticated information theory and competing to improve their

performance (Sutton, "The Bitter Success")[39]. This finding has two implications for future neuroscience research.

First, with the relative ease of creating artificial circuits imitating the brain, neuroscience research combining experiments and artificial neural networks will become increasingly prominent. Conventional circuits designed by computational neuroscientists were based on sophisticated theories and were often esoteric to other neuroscientists, but the advent of CNNs and transformers has made it possible to compare the brain with simpler (albeit large-scale) artificial neural circuits. As a result, the use of artificial neural networks is becoming an essential approach across neuroscience research. That said, the information processing in AI circuits remains a black box, and it is largely unclear what specific steps and intermediate stages are taken to process information. As we move forward, the rich reservoir of neuroscience theories on computational mechanisms with sophisticated designs will play a crucial role in understanding the internal processing of AI circuits and brain-like circuits.

Second, the emergence of immediate adaptability to novel tasks through the scaling up of circuits and training data in AI will raise awareness of the importance of circuit scale in neuroscience research. For example, this AI finding suggests that to elucidate sophisticated human-characteristic processing, human brain circuits, which are far larger than those of mice or monkeys, need to be examined. On the other hand, to elucidate the design principles of brain circuits, it is beneficial to study non-human brains, which are similar in design to the human brain, albeit on a smaller scale. Although the view that intelligence lies not in cleverly designed circuits but in large circuits has not been given much weight in neuroscience, awareness of the scale of circuits will be essential for future research on human intelligence.

References

1 Zhuang, C. *et al.* Unsupervised neural network models of the ventral visual stream. *Proc Natl Acad Sci U S A* **118**, doi:10.1073/pnas.2014196118 (2021).

2 LeCun, Y., Bengio, Y. & Hinton, G. Deep learning. *Nature* **521**, 436-444, doi:10.1038/nature14539 (2015).

3 Doya, K. Complementary roles of basal ganglia and cerebellum in learning and motor control. *Curr Opin Neurobiol* **10**, 732-739, doi:10.1016/s0959-4388(00)00153-7 (2000).

4 Olshausen, B. A. & Field, D. J. Emergence of simple-cell receptive field properties by learning a sparse code for natural images. *Nature* **381**, 607-609, doi:10.1038/381607a0 (1996).

5 Li, N. & DiCarlo, J. J. Unsupervised natural experience rapidly alters invariant object representation in visual cortex. *Science* **321**, 1502-1507, doi:10.1126/science.1160028 (2008).

6 Lillicrap, T. P., Santoro, A., Marris, L., Akerman, C. J. & Hinton, G. Backpropagation and the brain. *Nature reviews* **21**, 335-346, doi:10.1038/s41583-020-0277-3 (2020).

7 Blakemore, C. & Cooper, G. F. Development of the brain depends on the visual environment. *Nature* **228**, 477-478, doi:10.1038/228477a0 (1970).

8 Hinton, G. E. & Salakhutdinov, R. R. Reducing the dimensionality of data with neural networks. *Science* **313**, 504-507, doi:10.1126/science.1127647 (2006).

9 Bengio, Y., Lamblin, P., Popovici, D. & Larochelle, H. Greedy layer-wise training of deep networks. *Advances in neural information processing systems* **19** (2006).

10 Saxe, A. M. *Deep Linear Neural Networks: A Theory of Learning In the Brain and Mind* The Degree of Doctor of Philosophy thesis, Stanford University, (2015).

11 Fukushima, K. Neocognitron: A self-organizing neural network model for a mechanism of pattern recognition unaffected by shift in position. *Biological cybernetics* **36**, 193-202 (1980).

12 Le, Q. V. *et al.* Building high-level features using large scale unsupervised learning. Report No. 1479903566, 8595-8598 (IEEE, 2012).

13 Mineault, P. *Is early vision like a convolutional neural net?*, <https://xcorr.net/2021/04/16/is-early-vision-like-a-convolutional-neural-net/> (2021).

14 Kornblith, S., Shlens, J. & Le, Q. V. Do better imagenet models transfer better? , 2661-2671 (2019).

15 Pan, S. J. & Yang, Q. A survey on transfer learning. *IEEE Transactions on knowledge and data engineering* **22**, 1345-1359 (2009).

16 Zeiler, M. D. & Fergus, R. Visualizing and Understanding Convolutional Networks. (2014).

17 Yosinski, J., Clune, J., Nguyen, A., Fuchs, T. & Lipson, H. Understanding neural networks through deep visualization. *arXiv preprint arXiv:1506.06579* (2015).

18 Olah, C., Mordvintsev, A. & Schubert, L. Feature visualization. *Distill* **2**, e7 (2017).

19 Kitazawa, S. *Introduction to AI for medical doctors and students (Ishi igakusei no tameno AI nyuumon)*. (Chugai-igaku, 2020).

20 Goodfellow, I., Bengio, Y. & Courville, A. *Deep Learning*. (The MIT press, 2016).

21 Devlin, J., Chang, M.-W., Lee, K. & Toutanova, K. Bert: Pre-training of deep bidirectional transformers for language understanding. *arXiv preprint arXiv:1810.04805* (2018).

22 He, K. *et al.* Masked autoencoders are scalable vision learners. 16000-16009 (2022).

23 Okanohara, D. *The forefront of AI technology ("AI gijyutsu no saizensen" in Japanese)*. (Nikkei BP, 2022).

24 Lee, H., Grosse, R., Ranganath, R. & Ng, A. Y. Convolutional deep belief networks for scalable unsupervised learning of hierarchical representations. 609-616 (2009).

25 Yamins, D. L. *et al.* Performance-optimized hierarchical models predict neural responses in higher visual cortex. *Proc Natl Acad Sci U S A* **111**, 8619-8624, doi:10.1073/pnas.1403112111 (2014).

26 Yamins, D. L. & DiCarlo, J. J. Using goal-driven deep learning models to understand sensory cortex. *Nat Neurosci* **19**, 356-365, doi:10.1038/nn.4244 (2016).

27 Saxe, A., Nelli, S. & Summerfield, C. If deep learning is the answer, what is the question? *Nat Rev Neurosci* **22**, 55-67, doi:10.1038/s41583-020-00395-8 (2021).

28 Schrimpf, M. *et al.* Brain-score: Which artificial neural network for object recognition is most brain-like? *BioRxiv*, 407007 (2018).

29 Konkle, T. & Alvarez, G. A. A self-supervised domain-general learning framework for human ventral stream representation. *Nat Commun* **13**, 491, doi:10.1038/s41467-022-28091-4 (2022).

30 Eslami, S. M. A. *et al.* Neural scene representation and rendering. *Science* **360**, 1204-1210, doi:10.1126/science.aar6170 (2018).

31 Frankle, J., Dziugaite, G. K., Roy, D. M. & Carbin, M. Stabilizing the lottery ticket hypothesis. *arXiv preprint arXiv:1903.01611* (2019).

32 Frankle, J., Dziugaite, G. K., Roy, D. & Carbin, M. Linear mode connectivity and the lottery ticket hypothesis. Report No. 2640-3498, 3259-3269 (2020).

33 Von Oswald, J. *et al.* Transformers learn in-context by gradient descent. Report No. 2640-3498, 35151-35174 (2023).

34 Garg, S., Tsipras, D., Liang, P. S. & Valiant, G. What can transformers learn in-context? a case study of simple function classes. *Advances in Neural Information Processing Systems* **35**, 30583-30598 (2022).

35 Hassibi, B., Stork, D. G. & Wolff, G. J. Optimal brain surgeon and general network pruning. Report No. 0780309995, 293-299 (IEEE, 1993).

36 Han, S., Pool, J., Tran, J. & Dally, W. Learning both weights and connections for efficient neural network. *Advances in neural information processing systems* **28** (2015).

37 LeCun, Y., Denker, J. S. & Solla, S. A. in *Neural Information Processing Systems.*

38 Li, H., Kadav, A., Durdanovic, I., Samet, H. & Peter Graf, H. Pruning Filters for Efficient ConvNets. (2016).

39 Sutton, R. S. *The Bitter Lesson*, <http://www.incompleteideas.net/IncIdeas/BitterLesson.html> (2019).
